# Genomic and Transcriptomic Profiling of Brain Metastases

**DOI:** 10.3390/cancers13225598

**Published:** 2021-11-09

**Authors:** Christopher P. Wardell, Emilie Darrigues, Annick De Loose, Madison P. Lee, Murat Gokden, Issam Makhoul, Alan J. Tackett, Analiz Rodriguez

**Affiliations:** 1 Department of Biomedical Informatics, University of Arkansas for Medical Sciences, 4301 West Markham Street, Little Rock, AR 72205, USA; cpwardell@uams.edu; 2 Winthrop P. Rockefeller Cancer Institute, University of Arkansas for Medical Sciences, 4301 West Markham Street, Little Rock, AR 72205, USA; edarrigues@uams.edu (E.D.); makhoulissam@uams.edu (I.M.); AJTackett@uams.edu (A.J.T.); 3 Department of Neurosurgery, University of Arkansas for Medical Sciences, 4301 West Markham Street, Little Rock, AR 72205, USA; adeloose@uams.edu (A.D.L.); mplee@uams.edu (M.P.L.); 4 Department of Pathology, University of Arkansas for Medical Sciences, 4301 West Markham Street, Little Rock, AR 72205, USA; gokdenmurat@uams.edu; 5 Department of Oncology, University of Arkansas for Medical Sciences, 4301 West Markham Street, Little Rock, AR 72205, USA; 6 Department of Biochemistry, University of Arkansas for Medical Sciences, 4301 West Markham Street, Little Rock, AR 72205, USA

**Keywords:** bioinformatics, genomics, transcriptomics, brain metastases

## Abstract

**Simple Summary:**

Brain metastases (BM) are the most common brain tumors in adults and are the main cause of cancer-associated death. Omics analysis of BM will allow for a better understanding of metastatic progression, prognosis and therapeutic targeting. In this study, BM samples underwent comprehensive molecular profiling with genomics and transcriptomics. Mutational signatures suggested that most mutations were gained prior to metastasis. A novel copy number event centered around the MCL1 gene was found in 75% of all samples. Transcriptomics revealed that melanoma BM formed a distinct cluster in comparison to other subtypes. Poor survival correlated to self-identified black race and absence of radiation treatment but not molecular profiles. These data identify potential new drivers of brain metastatic progression, implicate that melanoma BM are distinctive and likely responsive to unique therapies, and further investigation of sociodemographic and clinical features are needed in BM cohorts.

**Abstract:**

Brain metastases (BM) are the most common brain tumors in adults occurring in up to 40% of all cancer patients. Multi-omics approaches allow for understanding molecular mechanisms and identification of markers with prognostic significance. In this study, we profile 130 BM using genomics and transcriptomics and correlate molecular characteristics to clinical parameters. The most common tumor origins for BM were lung (40%) followed by melanoma (21%) and breast (15%). Melanoma and lung BMs contained more deleterious mutations than other subtypes (*p* < 0.001). Mutational signatures suggested that the bulk of the mutations were gained before metastasis. A novel copy number event centered around the MCL1 gene was found in 75% of all samples, suggesting a broader role in promoting metastasis. Unsupervised hierarchical cluster analysis of transcriptional signatures available in 65 samples based on the hallmarks of cancer revealed four distinct clusters. Melanoma samples formed a distinctive cluster in comparison to other BM subtypes. Characteristics of molecular profiles did not correlate with survival. However, patients with self-identified black race or those who did not receive radiation correlated with poor survival. These data identify potential new drivers of brain metastatic progression. Our data also suggest further investigation of sociodemographic and clinical features is needed in BM cohorts.

## 1. Introduction

Brain metastases (BM) are the most common brain tumors in adults and occur in up to 40% of all cancer patients. Patients with BM have a poor prognosis with a median survival of 3 to 27 months after diagnosis [1,2]. The primary tumors with the highest incidence of brain metastases include lung (40–50%), breast (15–25%), and melanoma (5–20%) [3]. Treatment of BM entails a combination of radiation with or without surgery and more recently targeted agents, and/or immunotherapy [4,5,6]. Prediction of which patients will respond to selective therapy is limited. The application of omics data to better understand clinical features of BM is in its infancy and typically involves a single omics approach [7,8,9,10,11]. Multi-omics approaches in cancer allow for understanding molecular mechanisms and identification of markers of diagnostic/prognostic significance [9,12].

Genomic characterization of brain metastases and matched primary tumors identified that in 53% of samples, clinically informative and potentially actionable alterations were present in BM [13]. Epigenetic profiling with DNA methylomes and gene expression profiles of BM can identify tissue of origin as well as therapeutically relevant subtypes [14,15]. These data support the utility of multi-omics profiling of BM in order to analyze molecular profiles, stratify patients, and develop precision medicine approaches [12,16,17,18].

A main limitation to studying BM is that not all lesions qualify for surgical resection, limiting the ability to perform robust molecular characterization [19,20,21,22]. Patients typically present with more than one BM (up to 80%) or more than three BM (up to 50%). Surgery, and thus tissue procurement, has limited efficacy in most patients with multiple BM [23,24,25,26,27]. Despite this limitation, at our institution we prospectively perform next generation sequencing which includes a DNA cancer gene panel and bulk RNA transcriptome on all surgically resected brain tumors [28]. In this study we profile 130 BM (68 from our institution) from surgically resected samples and correlate molecular characteristics to clinical parameters.

## 2. Materials and Methods

### 2.1. Patient and Sample Selection

We retrospectively reviewed our prospectively acquired next generation sequencing brain tumor biobank after obtaining approval from our Institutional Review Board (IRB #249928 and 239292). We identified patients from 19–82 (years) who had a brain metastasis that was surgically resected, underwent sequencing analysis, and had clinical parameters available (Table S1). Matched samples from primary tumors were not available for the UAMS cohort. We also analyzed BM data from dbGaP which consisted of 62 BM samples.

### 2.2. Sample Preparation and Sequencing

Paired normal and formalin fixed paraffin embedded tumor samples were processed and sequenced by Tempus to produce Tempus xT targeted panel data (Chicago, IL, USA). A full list of the genes covered is given in Table S2. Tumor DNA was extracted from tumor tissue sections with tumor cellularity higher than 20% and proteinase K digestion was performed. Total nucleic acid extraction was performed with a Chemagic360 instrument using a source-specific magnetic bead protocol. Total nucleic acid was utilized for DNA library construction, while RNA was further purified by DNaseI digestion and magnetic bead purification. The nucleic acid was quantified by a Quant-iT picogreen dsDNA reagent Kit or Quant-iT Ribogreen RNA Kit (Life Technologies, Carlsband, CA, USA), and quality confirmed using a LabChip GX Touch HT Genomic DNA Reagent Kit or LabChip RNA High HT Pico Sensitivity Reagent Kit (PerkinElmer, Akron, OH, USA).

For DNA library construction, 100 ng of DNA for each tumor and normal sample was mechanically sheared to an average size of 200 base pairs using a Covaris ultrasonicator. The libraries were prepared using the KAPA Hyper Prep Kit. Briefly, DNA underwent enzymatic end-repair and A-tailing, followed by adapter ligation, bead-based size selection, and PCR. After library preparation, each sample was hybridized to a custom designed probe set. Recovery and washing of captured targets were performed using the SeqCap hybridization and wash kit. The captured DNA targets were amplified using the KAPA HiFi HotStart ReadyMix. The amplified target-captured libraries were sequenced on an Illumina HiSeq 4000 System.

For RNA library construction, 100 ng of RNA per tumor sample was fragmented with heat in the presence of magnesium to an average size of 200 base pairs. The RNA then underwent first strand cDNA synthesis using random primers, followed by combined second strand synthesis and A-tailing, adapter ligation, bead-based cleanup, and library amplification. After library preparation, samples were hybridized with the IDT xGEN Exome Research Panel. Target recovery was performed using Streptavidin-coated beads, followed by amplification using the KAPA HiFi Library Amplification Kit. The RNA libraries were sequenced to obtain approximately 65 million reads on an Illumina HiSeq 4000 System.

DNA samples were sequenced to a median depth of 1873 (range 1088–3241). There was no significant difference between the depths of different BM subtypes. The comparison data set was acquired from dbGaP under accession number phs000730.v1.p1. A total of 62 samples were selected representing paired normal and tumor whole exome sequencing data from metastatic brain tumors of different subtypes. A subset of 58 of these had an additional sample from the primary tumor.

### 2.3. Somatic Mutation Analysis

Reads were aligned to the reference genome GRCh37 using BWA-MEM [29] and variants called using Strelka2 [30]. Variants were filtered using FiNGS [31] and annotated using VEP [32]. Significantly mutated genes were determined using dNdSCV [33]. Analysis was performed using the UAMS data, comparison data and combined data sets, with a significance threshold of *q* < 0.1. Mutational signatures were determined using the R package NMF.

### 2.4. Copy Number Variation Analysis

Using aligned normal and tumor bam files as input, CNVKIT [34] was used to segment the data and GISTIC2 [35] was used to determine both broad and focal regions of copy number variation. Analysis was performed using the UAMS data, comparison data and combined data sets. A significance threshold of *q* < 0.1 was used and copy number events were only reported if significant in all three. Additional focal events from the validation data set were reported if in regions not covered by the Tempus xT panel and with *q* < 1 × 10^−4^.

### 2.5. RNA Analysis

Reads were aligned to the reference genome GRCh37 using BWA-MEM [29] and Stringtie [36] was used to generate transcript abundances.

Gene expression estimates were input into the cloud-based Almac proprietary analysis pipeline used for downstream calculation of signature scores and visual reporting for claraT V3.0.0 content (Almac Diagnostic Services, https://www.almacgroup.com/diagnostics/claratreport/, accessed on 15 March 2021). claraT is a unique software-driven solution, classifying biologically relevant gene expression signatures into a comprehensive easy-to-interpret report. Version 3.0.0 claraT Total mRNA Report, reports on 10 key cancer biologies (Immuno-Oncology (IO), Epithelial to Mesenchymal Transition (EMT), Angiogenesis, Proliferation, Cell Death, Genome Instability, Energetics, Inflammation, Immortality and Evading Growth) representing the hallmarks of cancer [37] by providing expression of 92 unique gene expression signatures, 100 single gene drug targets and 7337 single genes. These signatures are a mixture of publicly available expression signatures mined from the literature and proprietary signatures developed by Almac Diagnostics, and higher scores represent higher abundances of transcripts in these signatures. The method is similar to geneset enrichment analysis (GSEA). Values were scaled to between 0 and 1 and unsupervised hierarchical clustering using Ward’s method was performed using Euclidean distance as the distance metric to cluster samples.

### 2.6. Survival Analysis

Univariate and multivariate modelling was performed using survival data for the UAMS patients. Significant variables in the univariate model were used to build a multivariate Cox proportional hazards model.

## 3. Results

### 3.1. Patient Cohort

Basic demographics of our cohort are outlined in Table 1. Briefly, from 2018 to 2020, 68 BM from 68 patients were resected and underwent next generation sequencing at our institution (University of Arkansas for Medical Sciences, UAMS) as is routine within our brain tumor protocol [28]. The study was approved by the UAMS Institutional Review Board. The most common tumor origins for BM in our cohort were lung (40%) followed by melanoma (21%) and breast (15%). The remainder were from a diverse range of sites including renal, gynecologic and esophageal cancers. A full list of tumor origins is provided in Table S1. The median age was 58 years and composed of 50% males. We also integrated public data downloaded from dbGaP which was composed of 62 BM patients [13].

### 3.2. Somatic Mutations and Significantly Mutated Genes

In the UAMS set of 68 patients we detected a median of 69 SNVs (range 13–1062) and 17 indels (range 2–187). In absolute terms, the comparison data set of 62 patients contained more mutations, with a median of 434 SNVs (range 79–3057) and 19 indels (range 2–1521). When considered together, the UAMS data had a higher mutation rate per megabase of genomic DNA compared to the comparison set (median 24.8 SNVs/Mb versus 11.3 SNVs/Mb). However, when subtype was matched these differences were not significant. Melanoma had a high mutational burden and represented a much higher proportion of UAMS samples (14/68 compared to 2/62). We also found a linear correlation between the total number of mutations and the number of deleterious mutations in both the UAMS and comparison data sets, regardless of subtype origin (Pearson’s rho 0.94 and 0.95, *p* < 2.2 × 10^−16^). These data suggest that the UAMS and dbGaP data sets are comparable and that combining them and performing further analysis is a reasonable strategy.

**Figure 1:**
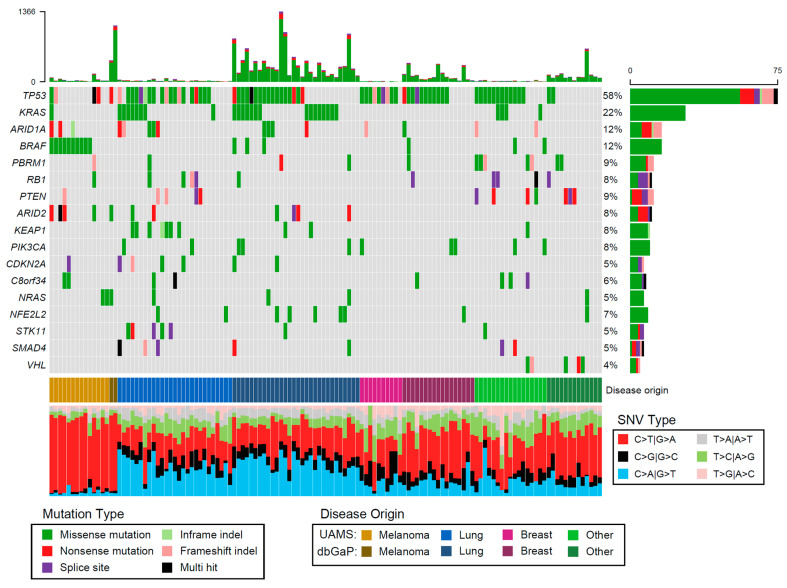
Oncoplot of frequently mutated genes in BM. The 17 significantly mutated genes were selected, and ordered by their overall frequency. Each column represents a single sample and each row a gene, with samples grouped by subtype. The barplot above and to the right represent the total number of deleterious mutations in each sample and the type of mutations detected in each gene, respectively. The stacked barplot at the bottom shows the contributions of each of the 6 mutation classes in each sample.

Melanoma and lung BMs contained more deleterious mutations than other subtypes (*p* < 0.001) (Figure 1), but not significantly more than one another, likely attributable to the highly mutagenic effects of ultraviolet radiation and tobacco use, respectively. These differences were driven by SNVs, as the number of indels was significantly lower in melanoma BMs compared to non-lung and breast samples (*p* = 0.005).

Seventeen genes were found to be significantly mutated (Figure 1), and the frequency of gene mutations were not significantly different between the UAMS and comparison data set. Significant genes included known pan-cancer tumor suppressors and oncogenes as well as more disease-specific genes, mostly restricted to the expected disease subtypes. These expectations were driven by mining public cancer genomics resources including the cancer genoma atlas (TCGA), catalogue of somatic mutations in cancer (COSMIC) and the Broad Institute’s TumorPortal, as well as the literature available in PubMed. For example, 9/11 BRAF V600E mutations were in melanoma samples, 9/10 KEAP1 mutations were in lung samples and 5/5 VHL mutations were in clear cell renal cancer samples. There was a significant correlation (Pearson’s rho 0.44, *p* = 1.33 × 10^−7^) between KEAP1 and STK11 mutations, with four samples containing both mutations.

The most commonly mutated gene was TP53, found in 58% of samples. In 89% of the samples, a mutation in at least one of the significantly mutated genes (median 2, range 0–7) was found. We found that 80% of all samples had at least one mutation or CNV affecting TP53, with 47% of all samples suffering from biallelic loss of TP53.

### 3.3. Mutational Signatures

**Figure 2:**
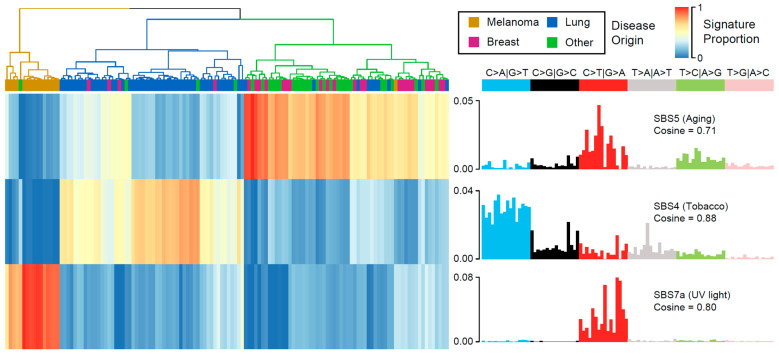
Mutational signatures detected in BM samples. Hierarchical clustering of the contribution of the three signatures suggests that most mutations were acquired before metastasis occurred, as they reflect the etiology of the primary disease (UV exposure for melanoma and tobacco exposure for lung cancers), with the remainder dominated by an age-related signature.

Different mutational processes act upon DNA, and each of these leaves a unique combination of mutation types. These mutational signatures can be used to determine the origin and timing of somatic mutations. Mutational signatures have been actively investigated for nearly a decade, but the central premise remains the same and now comprises some 60 single base substitution (SBS) signatures, 11 doublet-based signatures and 18 indel signatures [38,39]. Nonnegative matrix factorization (NMF) was used to determine mutational signatures and cosine similarity (CS) was used as measure of similarity to previously elucidated COSMIC signatures. CS is conceptually similar to Pearson correlation, with values closer to 1 denoting high similarity. We identified three SBS signatures with high similarity to those identified in COSMIC V3.2 (https://cancer.sanger.ac.uk/signatures/, accessed on 15 March 2021): SBS signature 5 (a broad-spectrum signature, possibly related to aging, CS 0.71), SBS signature 7a (ultraviolet light damage, CS 0.80), and SBS signature 4 (tobacco smoking, CS 0.88). The inferred etiology of these signatures is based on previous biochemistry studies in the literature. Hierarchical clustering of the contribution of these signatures to the mutational load of the BM samples resulted in clusters dominated by BMs from specific tissues (Figure 2), suggesting that the bulk of the mutations were gained before metastasis. This can be inferred because neither UV radiation nor tobacco smoke can directly cause mutations once cancer has metastasized from the skin or lungs to the brain. If additional mutational processes became active after metastasis, they did not produce enough mutations to be detected.

### 3.4. Copy Number Variation

**Figure 3:**
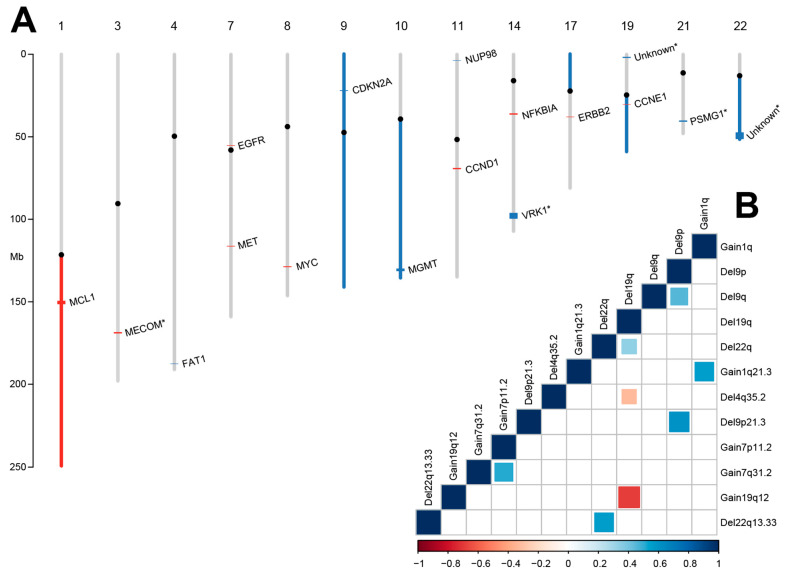
(**A**): Copy number events detected in BM. Red denotes gain and blue loss, with broad events coloring the entire arm and focal events represented using colored bars at the site of the events. Only affected chromosomes are shown. Candidate genes for focal events are labelled and events only identified in the dbGaP data set are labelled with an asterisk. Detailed tabulation is shown in Table 2 and Table 3. (**B**): Correlation plot of copy number events. Positive correlations with significant *p*-values after Bonferroni multiple testing correction (*p* < 1.7 × 10^−4^) were found for multiple pairs of features and were not restricted to specific subtypes. Pearson correlation is labelled with a color scheme from negative (red) to positive (blue) and larger colored squares represent smaller *p*-values.

We identified features at broad (chromosomal arm) and focal (megabases or less) scales (Figure 3A). At the broad scale, we found significant gain of chromosomes 1q (55%) and loss of chromosomes 9p (67%), 9q (53%), 10q (51%), 17p (58%), 19q (38%) and 22q (57%) (Table 2).

We identified 17 focal CNVs. The most common focal feature was a novel 2 Mb gain at 1q21.3 present in 75% of samples, regardless of disease origin. This region is centered upon MCL1, the only gene in the region covered by the targeted DNA panel. All other focal events occurring at >60% frequency were known tumor suppressors or oncogenes; gain8q24.21 (MYC), del9p21.3 (CDKN2A), and del10q26.2 (MGMT) in 68%, 67% and 60% of samples, respectively. There were no significant differences in frequency between the UAMS and dbGaP data sets. Of the dbGaP metastasis samples, 41/45 with gain1q21.3 had a matched primary sample, and 35/41 (85%) of these contained this lesion prior to metastasis. A data set consisting of cancer patients that did not develop brain metastases prior to death was not available, and brain metastases follow-up information is not available in public data repositories such as TCGA.

In general, we did not observe substantial variation in focal event frequency relative to the origin of disease, with three exceptions. Seventy percent (70%) of lung samples contained gain7p11.2 (EGFR) events (*p* = 0.004), 67% of breast samples contained gain11q13.3 (CCND1) events (*p* = 0.004) and 61% of lung samples contained gain14q13.2 (NFKBIA) events (*p* = 7 × 10^−5^).

We also identified five significant focal events in the comparison data set which we could not confirm in the UAMS data due to the genes not being covered. These included deletions of VRK1 and PSMG1 and gain of MECOM (Table 3).

We identified significant correlations between CNVs (Figure 3B). The majority were on the same chromosomes, but we also found a negative correlation between del4q35.2 (FAT1) and del19q (Pearson’s rho −0.33, *p* = 1.56 × 10^−4^) and a positive correlation between del19q and del22q (Pearson’s rho 0.33, *p* = 1.15 × 10^−4^).

### 3.5. RNA Sequencing

**Figure 4:**
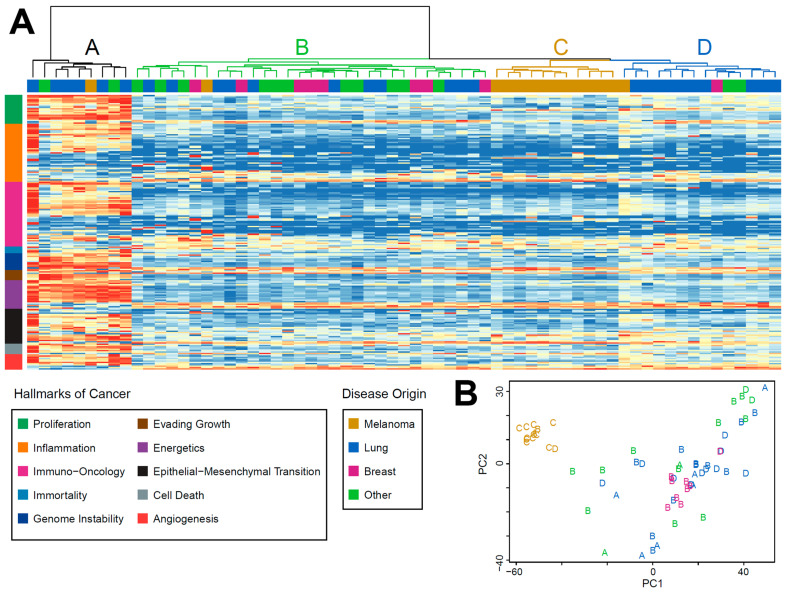
(**A**): Unsupervised hierarchical clustering of expression signatures based on the hallmarks of cancer in 65 UAMS BM samples. Scores are scaled between 0 (blue) and 1 (red), with higher values representing higher transcript abundances. Four clusters were formed. Notably, clusters C and D are predominantly melanoma and lung BM samples, respectively, and cluster A has a very transcriptionally active phenotype. (**B**): Principal component analysis of RNA sequencing data from 65 UAMS samples. Letters denote the RNA cluster from Figure 4A and colors denote the origin of disease. Melanoma BMs (orange letters) form a distinct cluster in both analyses, but the hallmark clusters are not replicated in the PCA, suggesting a genuine biological origin and not a batch effect or simple reflection of the origin of metastases. Signature names and references are detailed in order in Table S3.

Expression signatures based on the hallmarks of cancer were calculated for 65 of the UAMS samples using RNA sequencing data. Hierarchical clustering of these signatures produced four clusters (Figure 4A). Two of them appeared to be based on BM subtype. RNA cluster C was wholly composed of melanoma samples (11/14 total melanoma samples) and RNA cluster D was enriched for lung samples (*p* = 0.01), with 10/14 cluster members from this subtype. RNA cluster B was the largest cluster containing 31/65 samples and was composed of an assortment of subtypes. RNA cluster A was distinct from all others and scored highly across a range of cancer expression signatures, an inversion of the results in the other clusters. Other than disease origin for clusters C and D, we did not observe any significant correlation between any cluster and other molecular or clinical features.

Principal component analysis showed clustering based on the melanoma and breast subtypes but did not reproduce the patterns observed using the expression signatures, suggesting that the clusters formed by the expression signatures are neither batch effects nor driven solely by disease subtype (Figure 4B).

### 3.6. Survival Analysis

**Figure 5:**
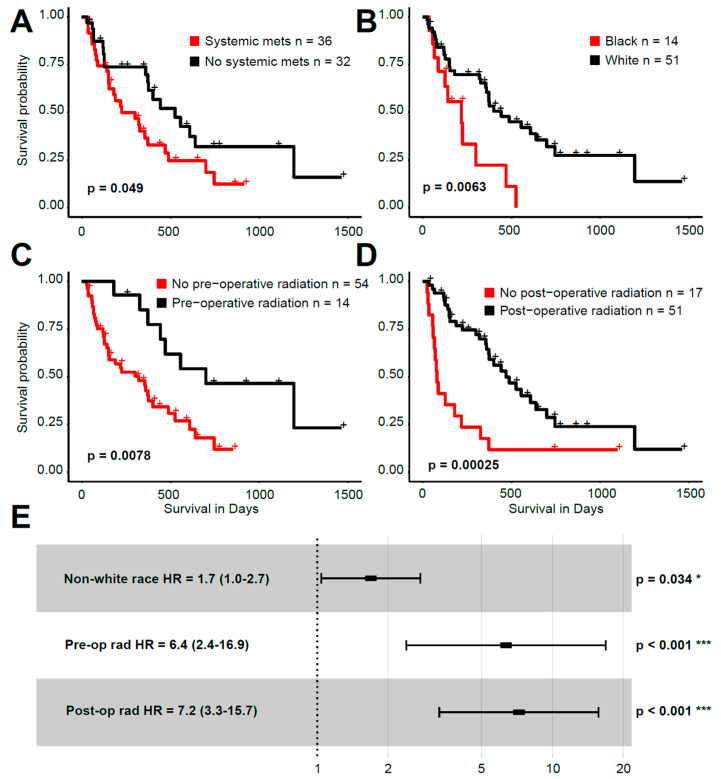
(**A**–**D**) Kaplan–Meier plots of overall survival for clinical features with univariate significance. (**E**): Forest plot of the hazard ratios (HR) for a multivariate Cox proportional hazards survival model (HR followed by 95% confidence interval in parentheses).

Overall survival analysis was performed using the UAMS patients. Genomic features (i.e., presence of certain mutations, SNVs, CNVs) and transcriptomic features (i.e., association with one of four distinct phenotypic clusters) did not correlate with overall survival (File S1). Four clinical features were significant in a univariate analysis (Figure 5A–D), with only the presence of systemic metastases not remaining significant in a multivariate analysis (Figure 5E). Clinical variables significant in the multivariate analysis were the self-reported race of the patients (HR = 1.7, CI 1.0–2.7, *p* = 0.034), pre-surgery radiation therapy (HR = 6.4, CI 2.4–16.9, *p* < 0.001) and post-surgery radiation therapy (HR = 7.2, CI 3.3–15.7, *p* < 0.001).

## 4. Discussion

Cancer therapy continues to evolve and overall survival has been significantly extended for many cancer types in high income countries [40]. However, BM remains a main cause of cancer-related mortality and the incidence is projected to increase over time [41]. The brain remains a sanctuary site for cancer as due to the brain–tumor barrier, few treatments are efficacious in the brain, limiting therapeutic options and prevention [42,43,44]. Many clinical trials for targeted and immune therapy excluded patients who had BM, but now these therapeutic strategies are recognized as possible treatments for certain patients [5,45,46,47,48,49]. Genomic characterization of BM has been useful in identification of potential driver and targetable mutations [13,50]. However, the recent results of the National Cancer Institute’s Molecular Analysis for Therapy Choice, a precision medicine trial based on DNA sequencing, demonstrated in a cohort of 4687 patients that only 17.8% of patients qualified to be assigned to therapy [51]. In a cohort of 500 cancer patients, genomic DNA profiling was able to identify potential targets for 29.6% of patients. This percentage increased to 43.4% with the integration of RNA sequencing and immune biomarkers [52]. In addition to the identification of potential therapeutic targets, multi-omics approaches for BM can be used to risk stratify patients and understand metastatic progression [53,54]. Therefore, in this study, we combined DNA and RNA sequencing to further characterize BM.

The internal and external data sets used in our study showed good concordance and similarity in regard to the genomic landscape. Through combining the data sets we were able to analyze 130 total BM samples. Overall, the TP53 gene had the most aberrations, which is consistent with previous literature identifying TP53 as the most common mutation in cancer [55,56,57]. At least one mutation was found in 58% of samples, and 58% had a deletion of 17p; in total, 47% of samples had complete biallelic inactivation of TP53. After TP53, the most commonly mutated genes included KRAS, ARID1A, and BRAF. BRAF was almost exclusively present in melanoma samples (Figure 1). All the significantly mutated genes have been previously identified as important in a variety of cancer types, with two exceptions. PBRM1 mutations have been associated with clear cell renal carcinoma [58], but in our BM data this mutation appeared in a broad spectrum of cases and infrequently with VHL mutations, suggesting it may have a broader role in promoting metastasis. Similarly, C8orf34 appeared in a variety of metastases and has been identified as a fusion gene partner of MET [59]. Although its function is unknown, it is a potential pan-cancer promoter of metastasis.

DNA mutation calling provided strong evidence that most mutations occurred prior to metastasis, as the NMF signatures clustered according to tissue origin, dominated by mutational processes such as tobacco or ultraviolet light damage (Figure 2). Further evidence of subtype-specific disease was found in the distribution of the genes mutated. Some genes were mostly restricted to certain subtypes; for example, BRAF mutations occurred mostly in melanoma, VHL mutations in kidney clear cell samples, and KEAP1 mutations were found in lung samples. Four samples contained both KEAP1 and STK11 mutations, which have been shown to indicate lung cancer patients [60].

Potentially the most interesting features were found in the copy number analysis. Our comparative external cohort had not been previously characterized for copy number analysis [13]. Copy number events tended not to be subtype-specific, with the exceptions of significant enrichments of lung samples containing gain7p11.2 (EGFR) and gain14q13.2 (NFKBIA) events and breast samples containing gain11q13.3 (CCND1) events (Figure 3A). Gain1q21.3, a novel copy number event centered around the MCL1 gene, was found in 75% of all samples. Interestingly, 85% of the dbGaP samples with gain1q21.3 also contained this lesion in a matched primary sample. Without a cohort of primary tumor samples in patients who were confirmed to not have developed brain metastases to compare against it is impossible to make definitive statements, but this suggests that gain of MCL1 could be an early driver of metastasis. MCL1 has been implicated in both the metastasis of clear cell renal carcinoma [61] and recurrence of breast cancer [62]. A combined 55% of samples had FAT1 lesions, predominantly as del4q35.2 (48%), with a minority of samples (14%) with mutations. This is interesting given that loss of function of FAT1 (by deletion or mutation) has been linked to promoting metastasis [63] and is a relatively uncommon cancer gene. We also found a significant negative correlation between del4q32.2 and del19q, suggesting that these regions are performing similar roles.

Transcriptome data were available for 65 BM samples, and we performed unsupervised hierarchical clustering analysis of transcriptional signatures based on the hallmarks of cancer which revealed four distinct clusters (Figure 4A). Two of these could be attributed to the tissue of origin, with clusters C and D dominated by melanoma and lung samples, respectively. Principal component analysis demonstrated that melanoma samples were the most similar to each other and formed a distinctive cluster in comparison to other BM subtypes (Figure 4B). From our gene expression clustering based on cancer hallmark signatures, clusters A and B were composed of various BM subtypes. Cluster A comprised nine patients and demonstrated increased expression in almost all gene expression pathways of the hallmarks of cancer (Figure 4A). We did not find any strong evidence related to other molecular or clinical features, and hypothesize that cluster A may be driven by epigenetic factors such as global DNA methylation changes, as this has been demonstrated to be a significant feature in BM, especially melanoma [14,64,65].

Lastly, we performed survival analysis on our cohort. A previous multi-omics analysis of BM patients demonstrated two molecular subtypes that correlated with survival and the development of distal brain metastases. With the utilization of proteomics and metabolomics, signaling pathways involved in the immune microenvironment as well as proliferation and migration were suggestive of the observed survival differences between groups [54]. We utilized both clinical, demographic, genomic and transcriptomic data in univariate and multivariate survival analysis. Interestingly, the presence of certain mutations, copy number events or belonging to specific gene expression cluster groups did not correlate with survival (File S1). Features that were statistically significant correlative to survival included presence of systemic metastases, self-identified race, and presence of pre-operative or post-operative radiation on univariate analysis (Figure 5A). On multivariate analysis, patients with self-identified black race and who did not receive radiation correlated with poor survival (Figure 5B). Race is a social construct but also is related to genetic ancestry [66]. Cancer disparities amongst racial/ethnic groups may be related to both social determinants of care and potential genetic and/or epigenetic etiologies [67,68]. Radiation therapy (RT) has been well established as an integral part of BM management and therefore it is not surprising that the absence of RT in the patient’s clinical course would lead to worse survival [19,69]. A limitation of our study is the cohort size, and an increase in the patient population in the future may allow us to define both clinical and genomic prognostic biomarkers further. We also only studied patients who underwent surgical resection and did not have matched primary tumors for a large proportion of the cohort studied. This limits us from understanding potential mechanisms of metastatic progression; nonetheless, our cohort remains one of the largest BM cohorts studied to date.

## 5. Conclusions

In our combined cohort of 130 patient BM samples, we identify features that are both disease-specific and novel ones that may be linked to metastasis, including a novel mutated gene (C8orf34), a novel gain of the gene MCL1 at 1q21.3 and a group of samples with an unusual transcriptional profile. Our comprehensive analysis allows for identification of prognostic biomarkers and potential therapeutic targets. Our analysis also demonstrates the need to integrate sociodemographic parameters in future studies as well.

## Figures and Tables

**Table 1:** Patient characteristics. The UAMS and dbGaP data sets were of similar sizes and compositions.

Feature (%)	All Patients (*n* = 130)	UAMS Patients (*n* = 68)	dbGaP Data Set (*n* = 62)
Sex:			
Female	76 (58%)	34 (50%)	42 (68%)
Male	54 (42%)	34 (50%)	20 (32%)
Age (mean, standard deviation, range)	57, 11.5, 19–82	58, 12.3, 19–82	56, 10.6, 35–80
Smoking	82 (63%)	41 (60%)	41 (66%)
Prior radiation	23 (18%)	13 (19%)	10 (16%)
Disease origin			
Breast	27 (21%)	10 (15%)	17 (27%)
Lung	57 (44%)	27 (40%)	30 (48%)
Melanoma	16 (12%)	14 (21%)	2 (3%)
Other	30 (23%)	17 (25%)	13 (21%)
Median survival	NA	372 days, 95% CI 299–555	NA

**Table 2:** Broad focal copy number events in BM.

Description	*q*-Value (Combined)	*q*-Value (UAMS)	*q*-Value (dbGaP)	Total Frequency (*n* = 130)	Frequency UAMS (*n* = 68)	Frequency dbGaP (*n* = 62)
Gain1q	1.69 × 10^−7^	4.13 × 10^−4^	3.84 × 10^−2^	55%	56%	53%
Del9p	3.66 × 10^−7^	1.03 × 10^−5^	3.35 × 10^−2^	67%	72%	58%
Del9q	6.15 × 10^−4^	5.14 × 10^−2^	1.98 × 10^−3^	53%	49%	60%
Del10q	4.12 × 10^−3^	5.14 × 10^−2^	1.20 × 10^−2^	51%	49%	61%
Del17p	7.16 × 10^−4^	4.22 × 10^−2^	1.98 × 10^−3^	58%	51%	73%
Del19q	2.78 × 10^−4^	5.14 × 10^−2^	3.35 × 10^−2^	38%	31%	50%
Del22q	1.11 × 10^−7^	5.14 × 10^−2^	5.05 × 10^−5^	57%	38%	74%

**Table 3:** Significant focal copy number events in BM, in order of overall frequency. Five features listed at the bottom of the table were only detectable in the comparison data set due to the coverage of the DNA capture used.

Description	Genomic Coordinates (hg19)	Size (Megabases)	RefSeq Genes in Window	Candidate Gene	*q*-Value (Combined)	*q*-Value (UAMS)	*q*-Value (Validation)	Total Frequency (*n* = 130)	Frequency UAMS (*n* = 68)	Frequency in dbGaP Data (*n* = 62)
Gain1q21.3	chr1:149459146–151435225	2.0	Many	MCL1	1.78 × 10^−7^	1.83 × 10^−4^	3.25 × 10^−3^	75%	78%	73%
Gain8q24.21	chr8:128600697–128904379	0.3	3	MYC	2.17 × 10^−3^	4.50 × 10^−5^	NA	68%	69%	68%
Del9p21.3	chr9:21853980–22169008	0.3	2	CDKN2A	3.76 × 10^−14^	2.73 × 10^−3^	6.30 × 10^−11^	67%	72%	61%
Del10q26.2	chr10:129878108–131344459	1.5	2	MGMT	2.33 × 10^−6^	7.26 × 10^−4^	NA	60%	65%	55%
Gain7q31.2	chr7:116187728–116418819	0.2	2	MET	3.62 × 10^−3^	3.89 × 10^−4^	NA	56%	57%	55%
Gain7p11.2	chr7:55220368–55419259	0.2	2	EGFR	7.99 × 10^−3^	6.91 × 10^−4^	NA	55%	60%	50%
Del11p15.4	chr11:3677960–3833468	0.2	4	NUP98	3.80 × 10^−4^	7.82 × 10^−2^	3.10 × 10^−3^	48%	50%	47%
Del4q35.2	chr4:187489123–187657360	0.2	1	FAT1	5.42 × 10^−7^	1.00 × 10^−2^	4.67 × 10^−4^	48%	60%	35%
Gain17q12	chr17:37856575–38035342	0.2	4	ERBB2	9.90 × 10^−15^	1.88 × 10^−9^	3.84 × 10^−7^	46%	54%	37%
Gain19q12	chr19:30313521–30466344	0.2	2	CCNE1	4.20 × 10^−2^	4.77 × 10^−4^	NA	42%	53%	31%
Gain14q13.2	chr14:35873861–36509568	0.6	4	NFKBIA	1.09 × 10^−5^	3.96 × 10^−3^	1.34 × 10^−2^	42%	47%	35%
Gain11q13.3	chr11:68944739–69588069	0.6	4	CCND1	4.19 × 10^−7^	1.79 × 10^−4^	NA	41%	43%	39%
Gain3q26.2	chr3:168496014–169105035	0.6	2	MECOM *	1.78 × 10^−7^	NA	5.62 × 10^−6^			79%
Del22q13.33	chr22:47340083–51304566	4.0	Many	Unknown *	7.93 × 10^−4^	NA	9.82 × 10^−4^			79%
Del19p13.3	chr19:1595678–2171104	0.6	Many	Unknown *	3.06 × 10^−4^	NA	1.80 × 10^−4^			73%
Del21q22.2	chr21:40162170–40831414	0.7	5	PSMG1 *	1.25 × 10^−6^	NA	7.37 × 10^−7^			63%
Del14q32.13	chr14:96158774–99640737	3.5	Many	VRK1 *	1.85 × 10^−14^	NA	2.52 × 10^−13^			45%

## Data Availability

Data from the UAMS cohort is available on dbGaP, accession number: phs002639.v1.p1. Data from the other clinical cohort analyzed in this study is also available on dbGaP, accession number: phs000730.v1.p1.

## Supplementary Materials

The following are available online at https://www.mdpi.com/article/10.3390/cancers13225598/s1. Table S1: Sample characteristics. Table S2: Genes covered by the Tempus xT targeted panel. Table S3: Signature names and references corresponding to Figure 4. File S1: Univariate survival analysis of all transcriptomic and genomic features. Sequencing data are available in dbGaP under accession number phs002639.v1.p1.
