# Supplementary material for: Genomic and Transcriptomic Profiling of Brain Metastases
Source: Cancers (Basel). 2021 Nov 9;13(22):5598. doi: 10.3390/cancers13225598 (PMC8615723; doi:10.3390/cancers13225598)
Supplement: Supplementary file 1 [file cancers-13-05598-s001.zip › supplementary/supplementary_file_1.pdf]

Supplementary File 1

Univariate overall survival analysis for transcriptomic groups, significantly mutated genes (siggenes) and copy number variants (CNVs), both broad and focal. Analysis performed using the R package "survival" and plots produced using the R package "survminer". Although two genes (NFE2L2 and SMAD4) had significant survival effects, they are not reported in the manuscript for two reasons. Firstly, the number of mutants with available survival data is relatively small and secondly no multiple testing correction has been applied, which would greatly affect the pvalues and make them non-significant.

Feature	Class	Mutants	Nonmutants	pvalue
RNA clusters	Transcription	NA	NA	0.64
TP53	Siggene	44	24	0.76
KRAS	Siggene	13	55	0.77
ARID1A	Siggene	11	57	0.81
BRAF	Siggene	12	56	0.89
PBRM1	Siggene	7	61	0.66
RB1	Siggene	8	60	0.81
PTEN	Siggene	9	59	0.11
ARID2	Siggene	6	62	0.68
KEAP1	Siggene	8	60	0.79
PIK3CA	Siggene	5	63	0.88
CDKN2A	Siggene	5	63	0.83
C8orf34	Siggene	7	61	0.76
NRAS	Siggene	3	65	0.24
NFE2L2	Siggene	2	66	0.016
STK11	Siggene	6	62	0.25
SMAD4	Siggene	5	63	0.048
VHL	Siggene	2	66	0.23
gain1q	Broad CNV	39	29	0.5
del9p	Broad CNV	62	6	0.33
del9q	Broad CNV	57	11	0.53
del10q	Broad CNV	58	10	0.13
del17p	Broad CNV	54	14	0.53
del19q	Broad CNV	32	36	0.4
del22q	Broad CNV	41	27	0.06
Gain1q21.3	Focal CNV	53	15	0.49
Gain8q24.21	Focal CNV	47	21	0.72
Del9p21.3	Focal CNV	49	19	0.47
Del10q26.2	Focal CNV	44	24	0.78
Gain7q31.2	Focal CNV	39	29	0.4
Gain7p11.2	Focal CNV	41	27	0.55
Del4q35.2	Focal CNV	41	27	0.2
Del11p15.4	Focal CNV	34	34	0.92
Gain17q12	Focal CNV	37	31	0.31
Gain19q12	Focal CNV	36	32	0.51
Gain14q13.2	Focal CNV	32	36	0.14
Gain11q13.3	Focal CNV	29	39	0.82

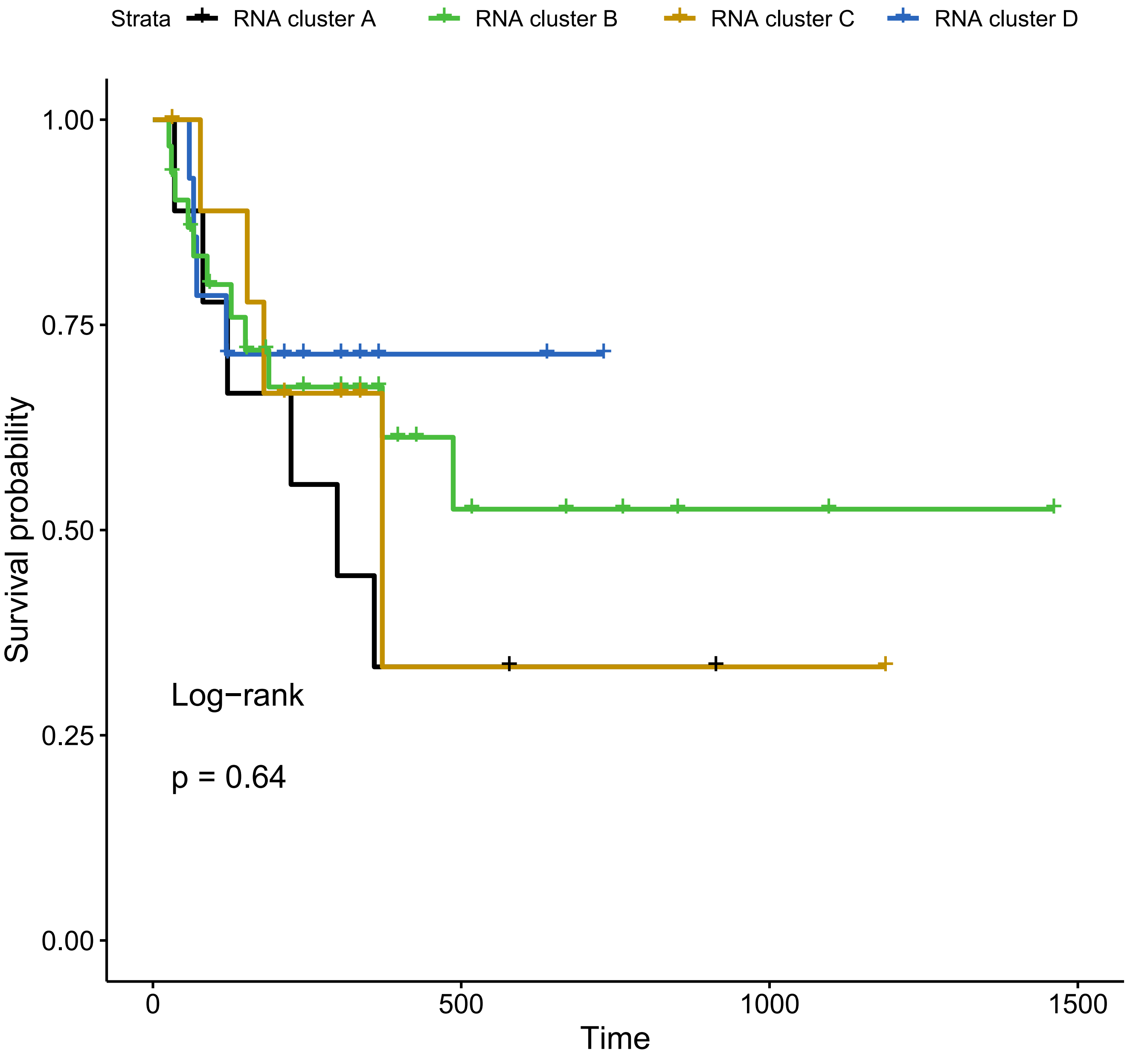

Strata
RNA cluster A
RNA cluster B
RNA cluster C
RNA cluster D
Survival probability
1.00
0.75
0.50
0.25
0.00
Log−rank
p = 0.64
0
500
1000
1500
Time

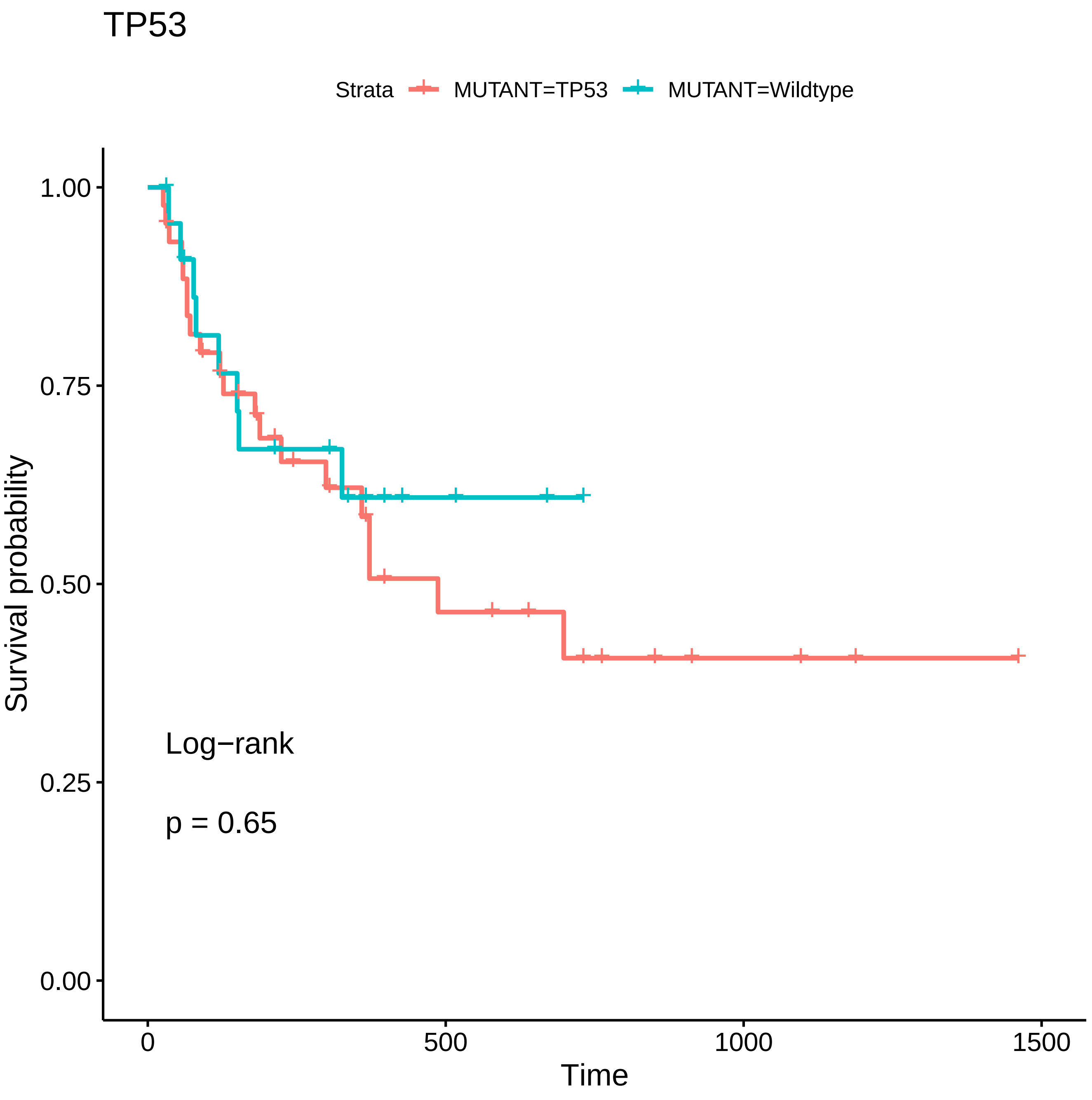
TP53
Strata
MUTANT=TP53
MUTANT=Wildtype
Survival probability
1.00
0.75
0.50
0.25
0.00
0
500
1000
1500
Time
Log−rank
p = 0.65

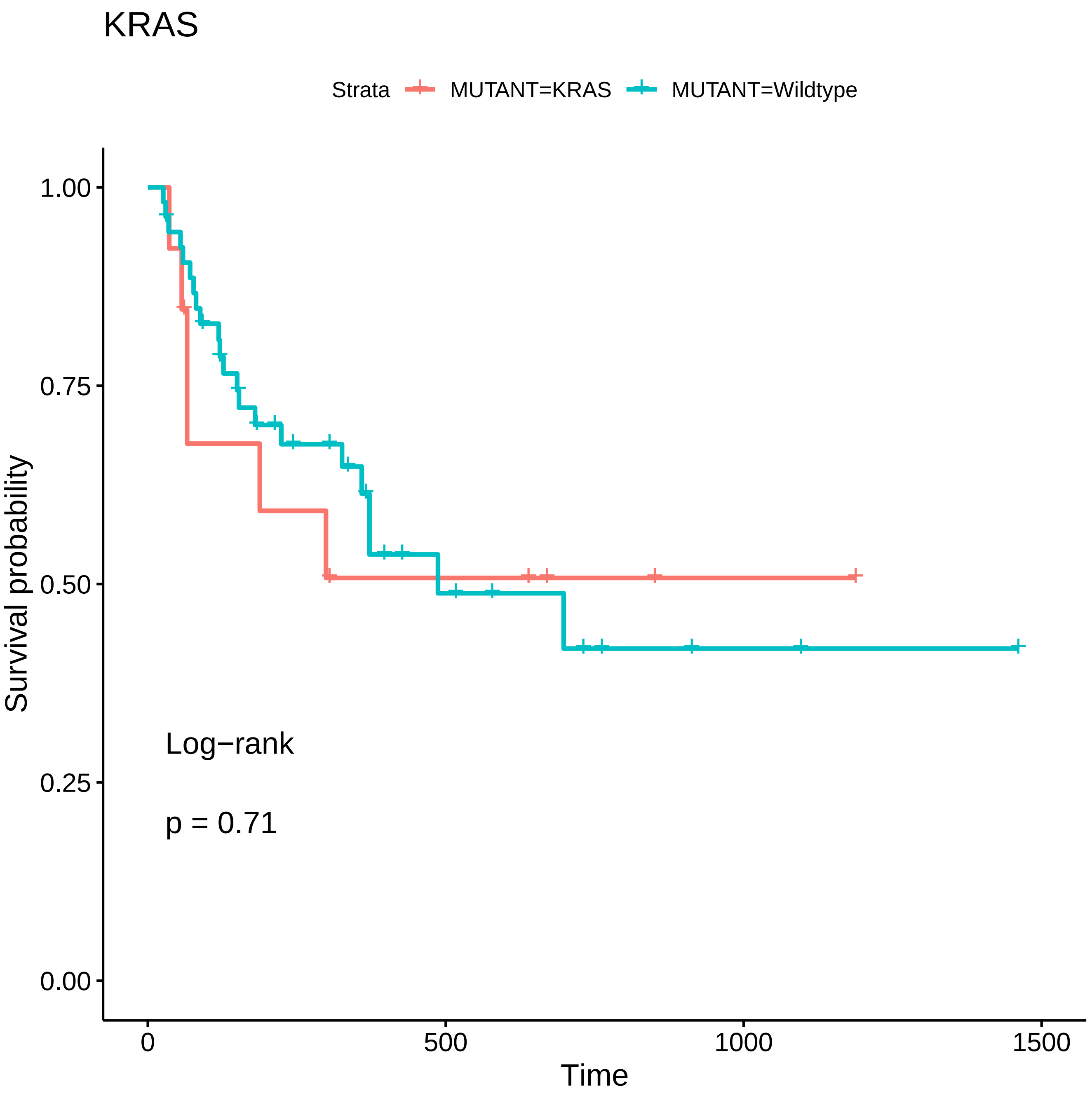

KRAS
Strata
MUTANT=KRAS
MUTANT=Wildtype
1.00
0.75
0.50
0.25
0.00
Survival probability
Log−rank
p = 0.71
0
500
1000
1500
Time

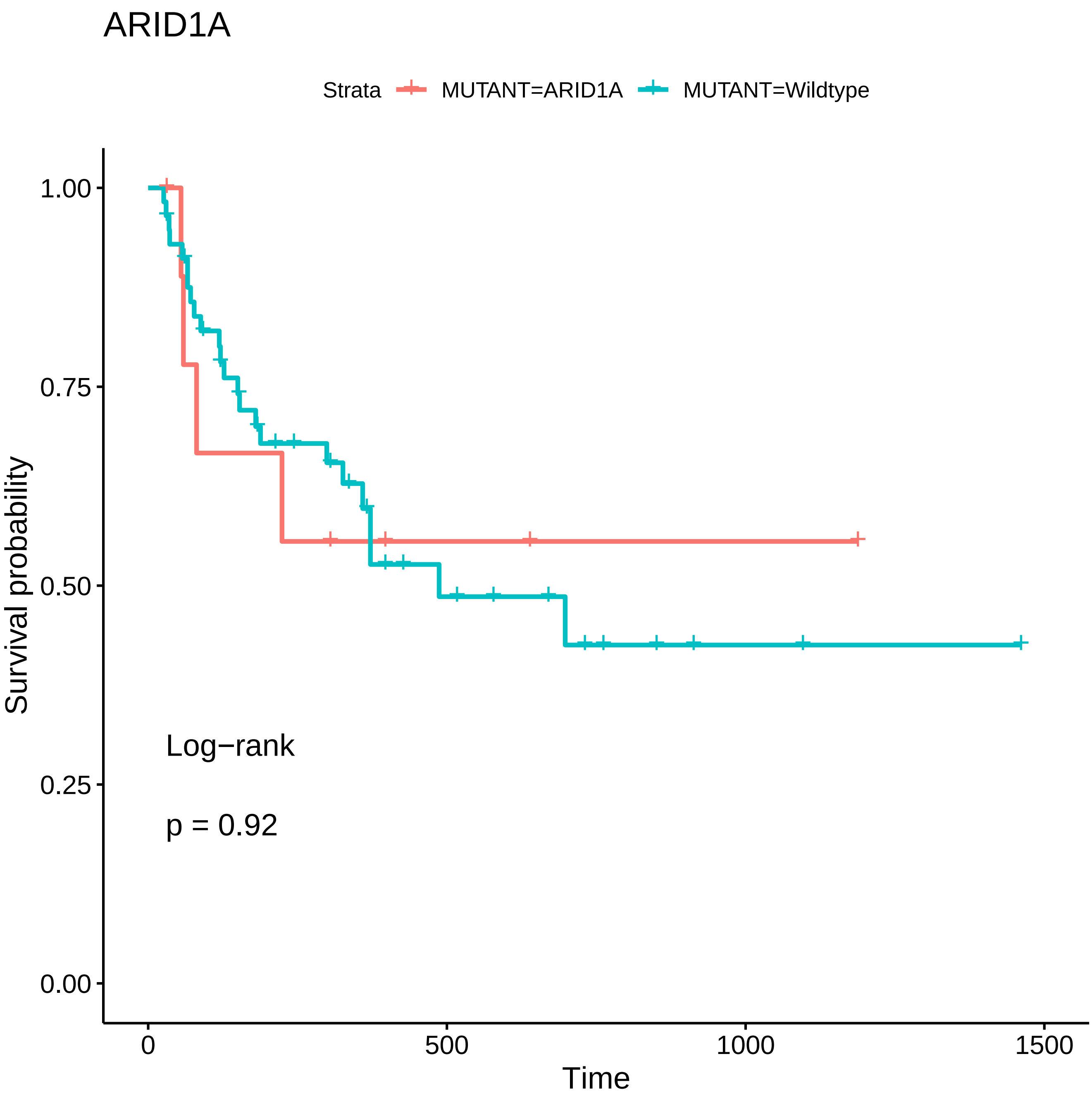

ARID1A
Strata
MUTANT=ARID1A
MUTANT=Wildtype
Survival probability
1.00
0.75
0.50
0.25
0.00
Log−rank
p = 0.92
0
500
1000
1500
Time

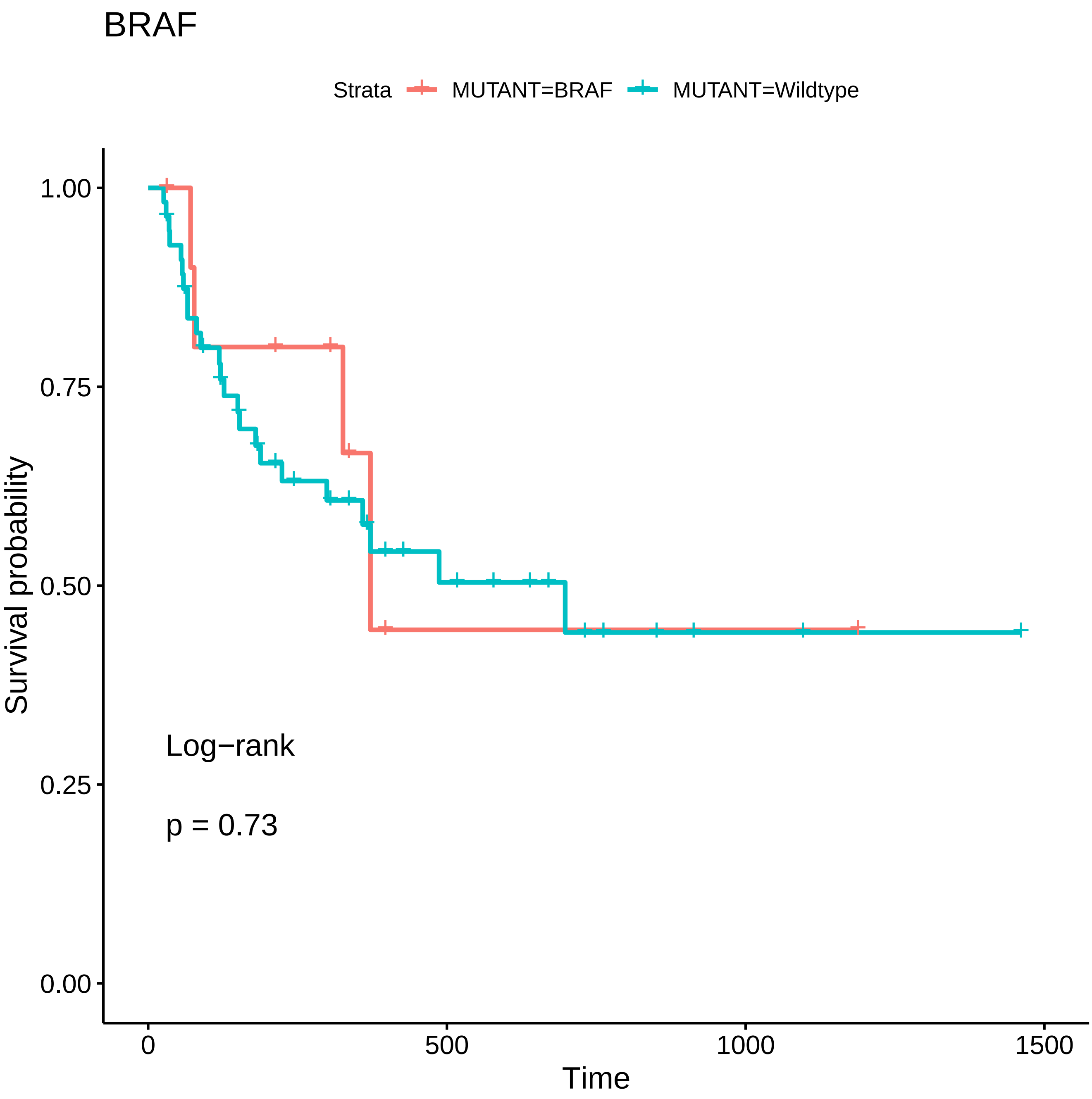
BRAF
Strata
MUTANT=BRAF
MUTANT=Wildtype
Survival probability
1.00
0.75
0.50
0.25
0.00
Log−rank
p = 0.73
0
500
1000
1500
Time

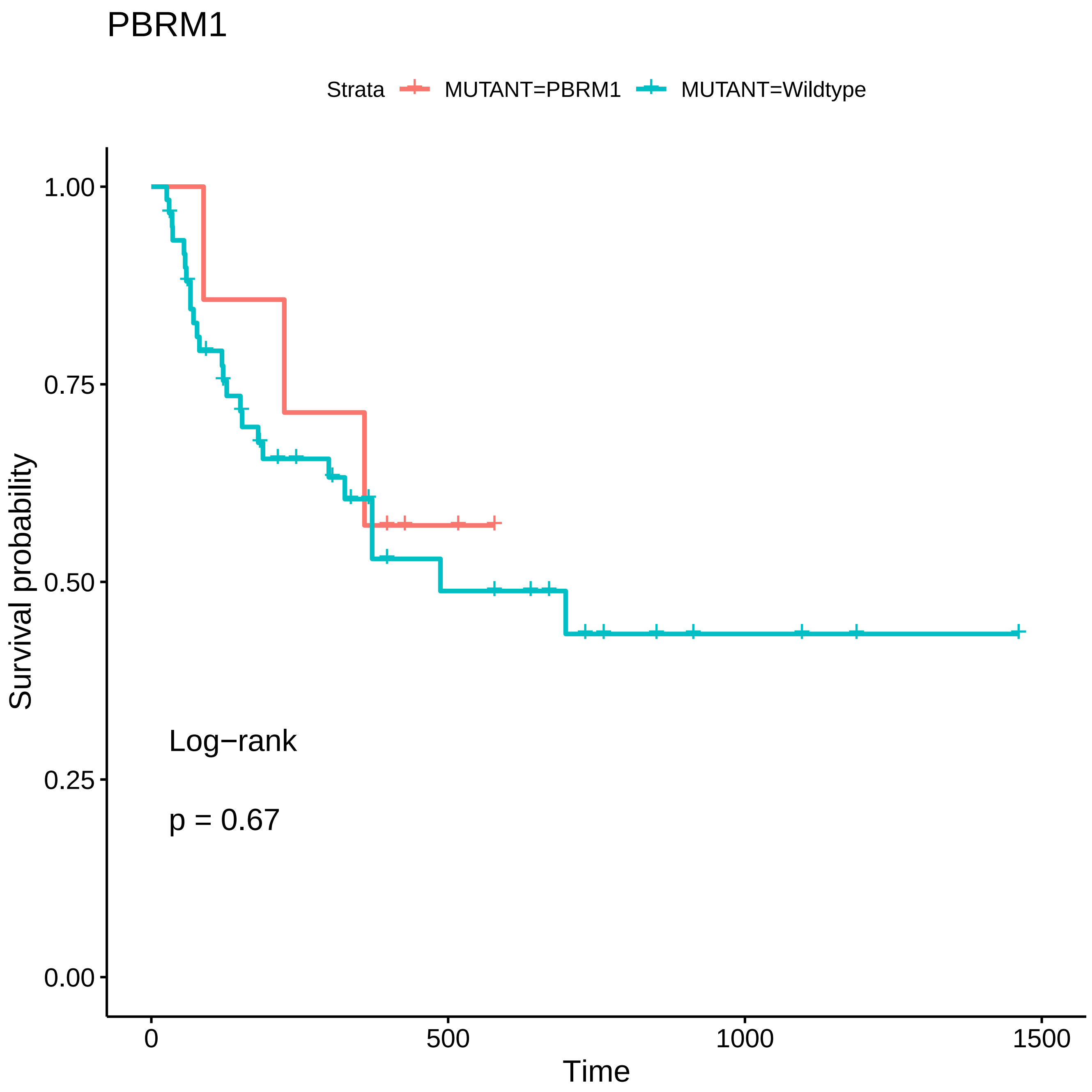
PBRM1
Strata
MUTANT=PBRM1
MUTANT=Wildtype
1.00
0.75
0.50
0.25
0.00
Survival probability
Log−rank
p = 0.67
0
500
1000
1500
Time

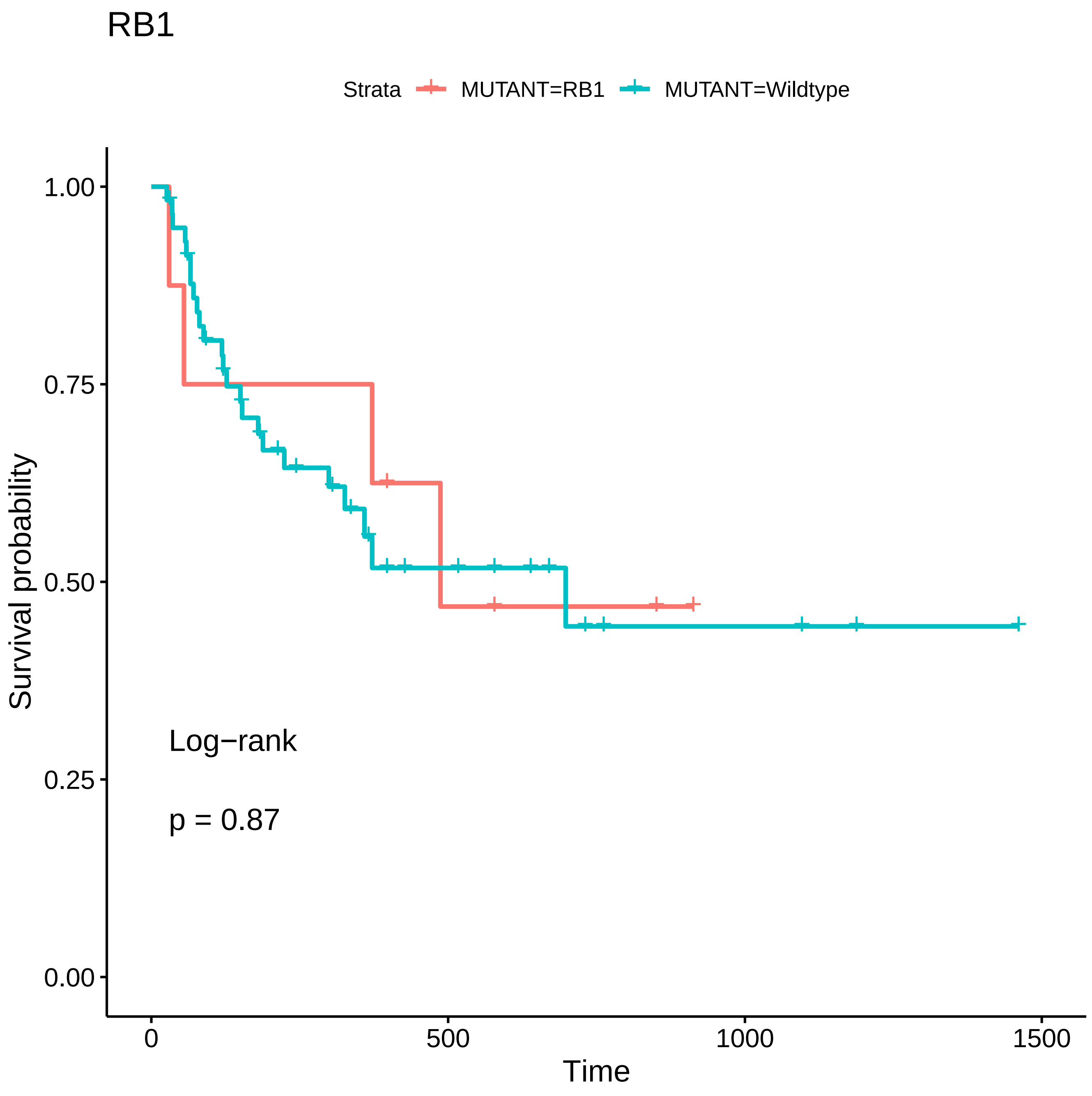

RB1
Strata
MUTANT=RB1
MUTANT=Wildtype
Survival probability
1.00
0.75
0.50
0.25
0.00
Log−rank
p = 0.87
0
500
1000
1500
Time

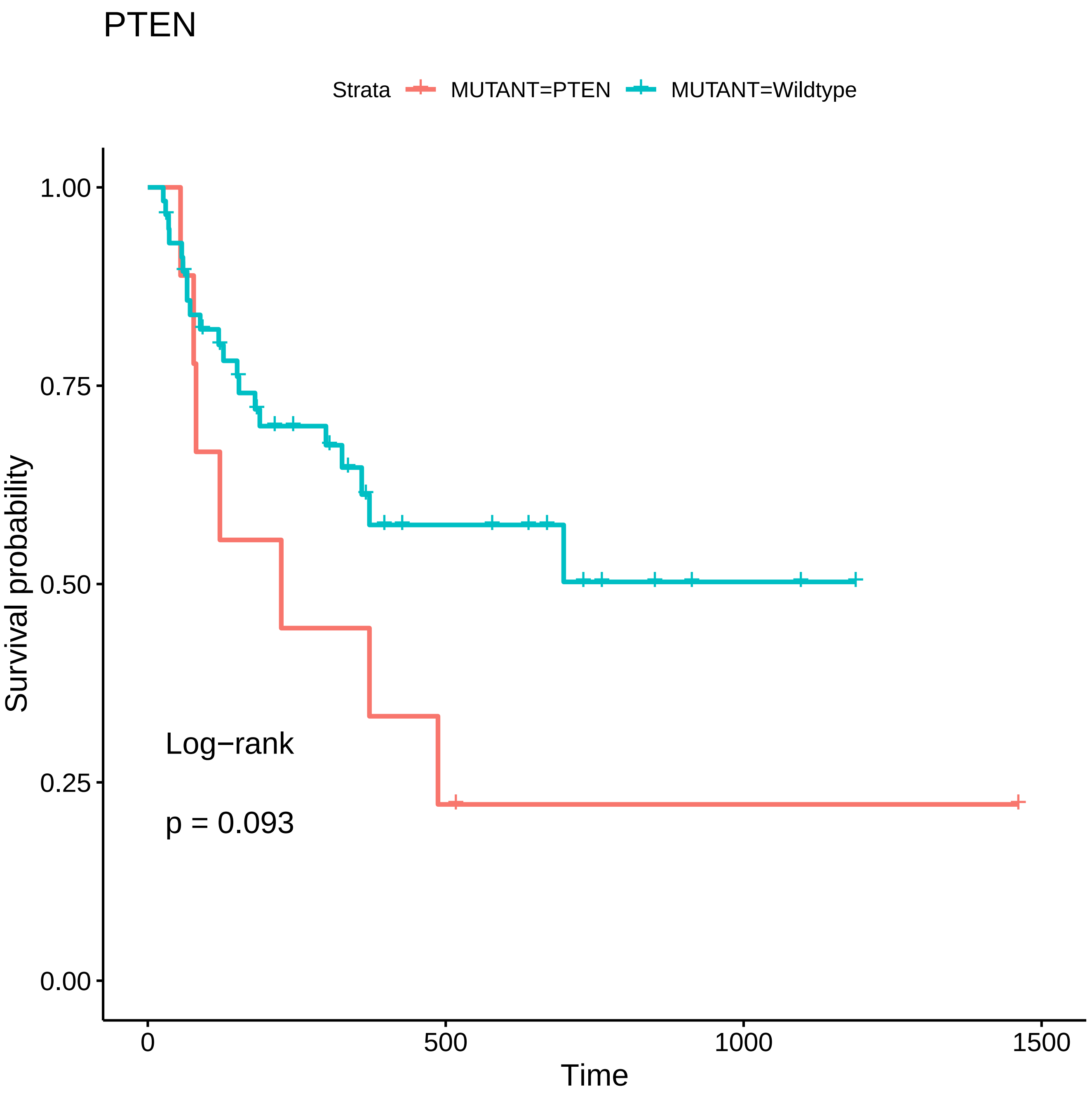
PTEN
Strata
MUTANT=PTEN
MUTANT=Wildtype
1.00
0.75
0.50
0.25
0.00
Survival probability
Log−rank
p = 0.093
0
500
1000
1500
Time

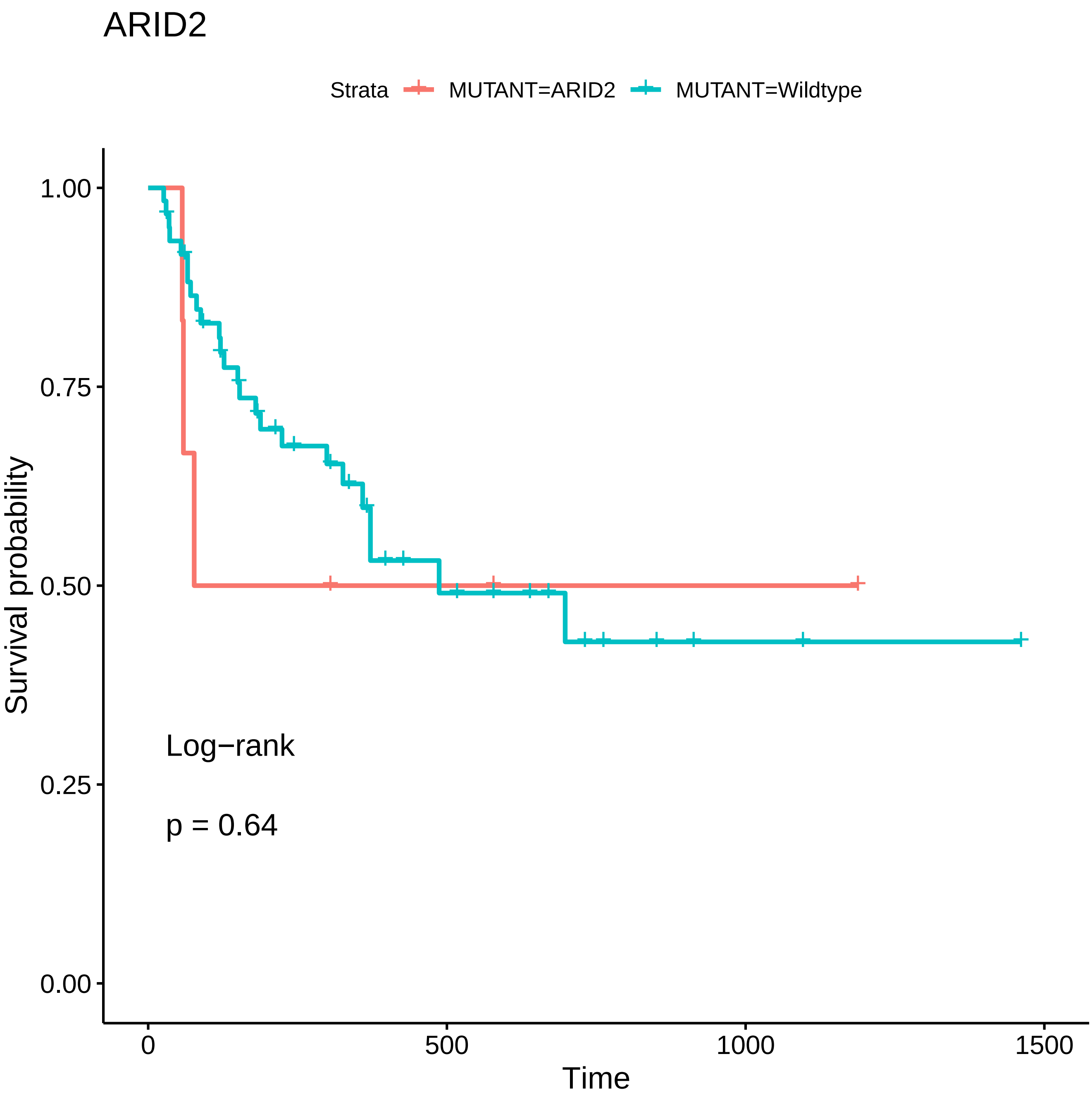

ARID2
Strata
MUTANT=ARID2
MUTANT=Wildtype
1.00
0.75
0.50
0.25
0.00
Survival probability
Log−rank
p = 0.64
0
500
1000
1500
Time

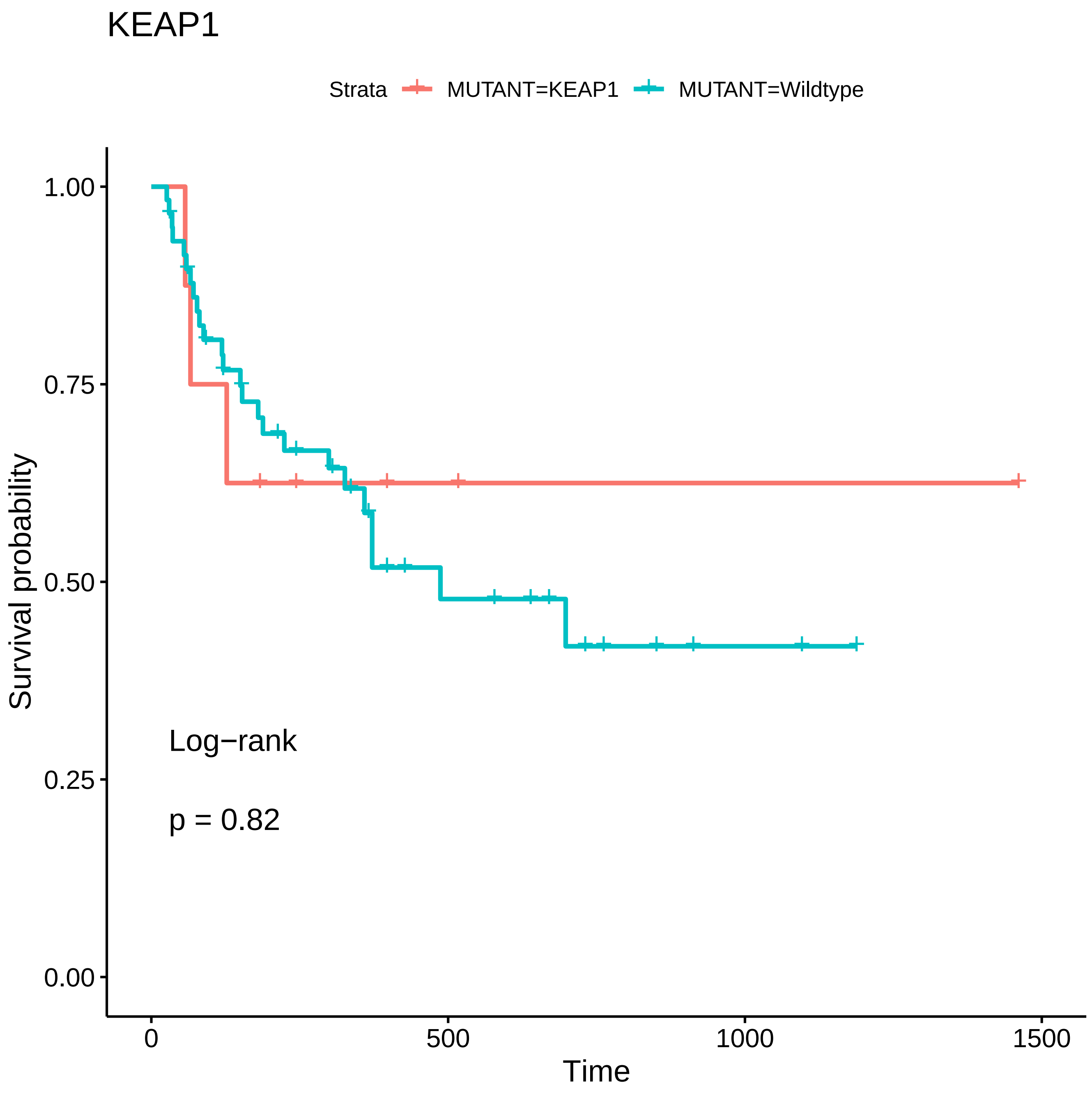
KEAP1
Strata
MUTANT=KEAP1
MUTANT=Wildtype
Survival probability
1.00
0.75
0.50
0.25
0.00
Log−rank
p = 0.82
0
500
1000
1500
Time

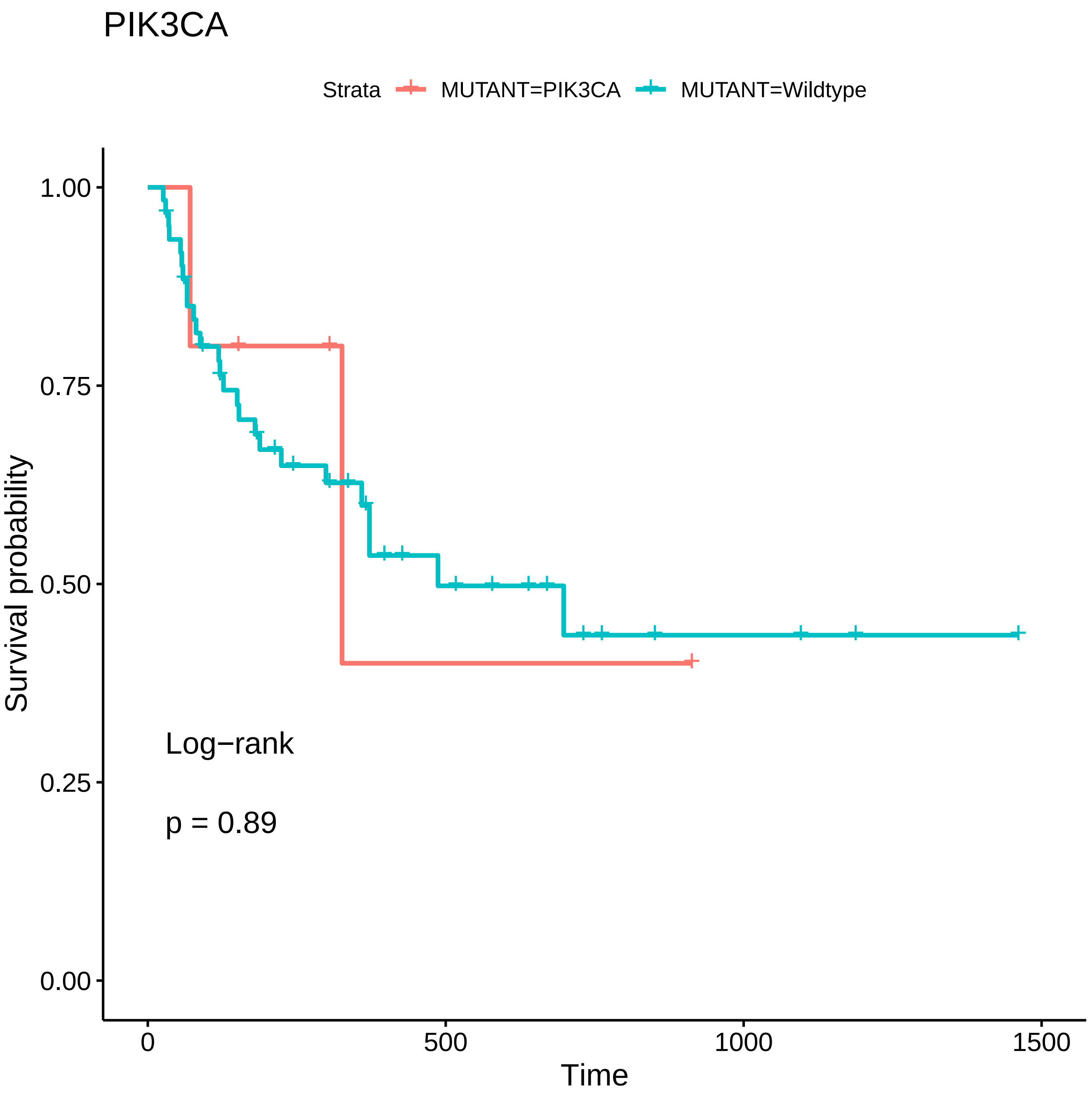
PIK3CA
Strata
MUTANT=PIK3CA
MUTANT=Wildtype
Survival probability
1.00
0.75
0.50
0.25
0.00
Log−rank
p = 0.89
0
500
1000
1500
Time

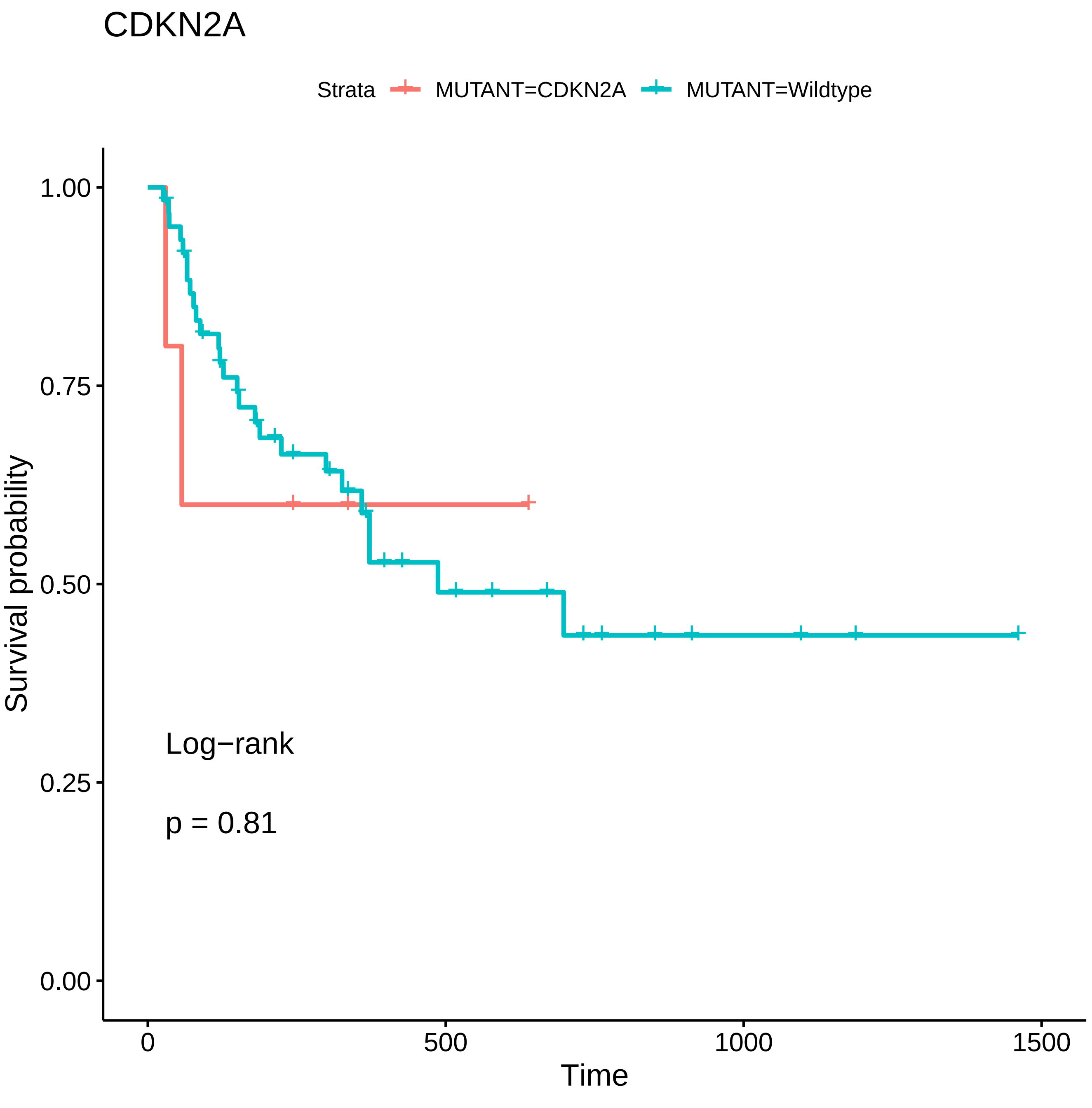
CDKN2A
Strata
MUTANT=CDKN2A
MUTANT=Wildtype
1.00
0.75
0.50
0.25
0.00
Survival probability
Log−rank
p = 0.81
0
500
1000
1500
Time

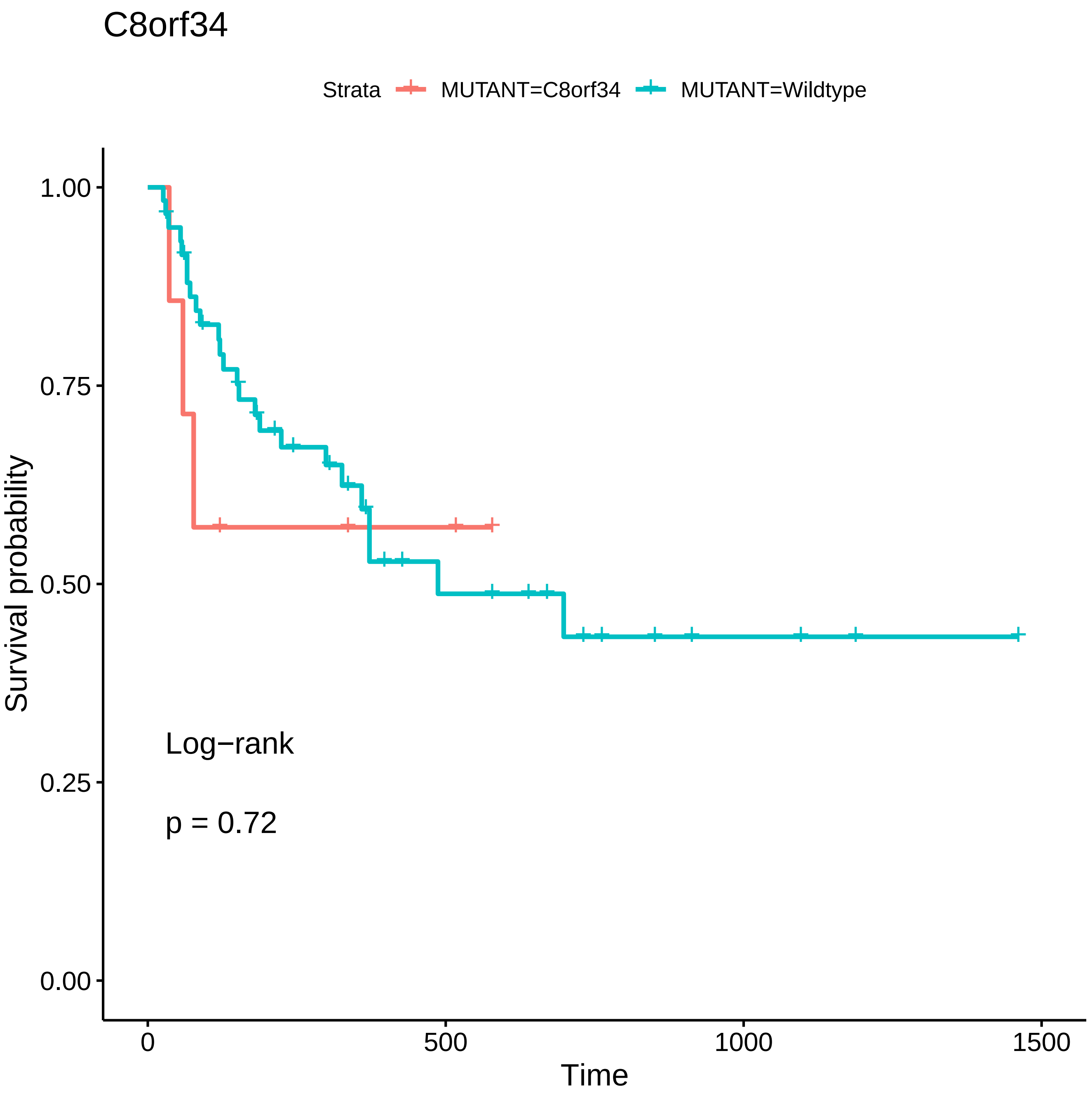

C8orf34
Strata
MUTANT=C8orf34
MUTANT=Wildtype
1.00
0.75
0.50
0.25
0.00
Survival probability
Log−rank
p = 0.72
0
500
1000
1500
Time

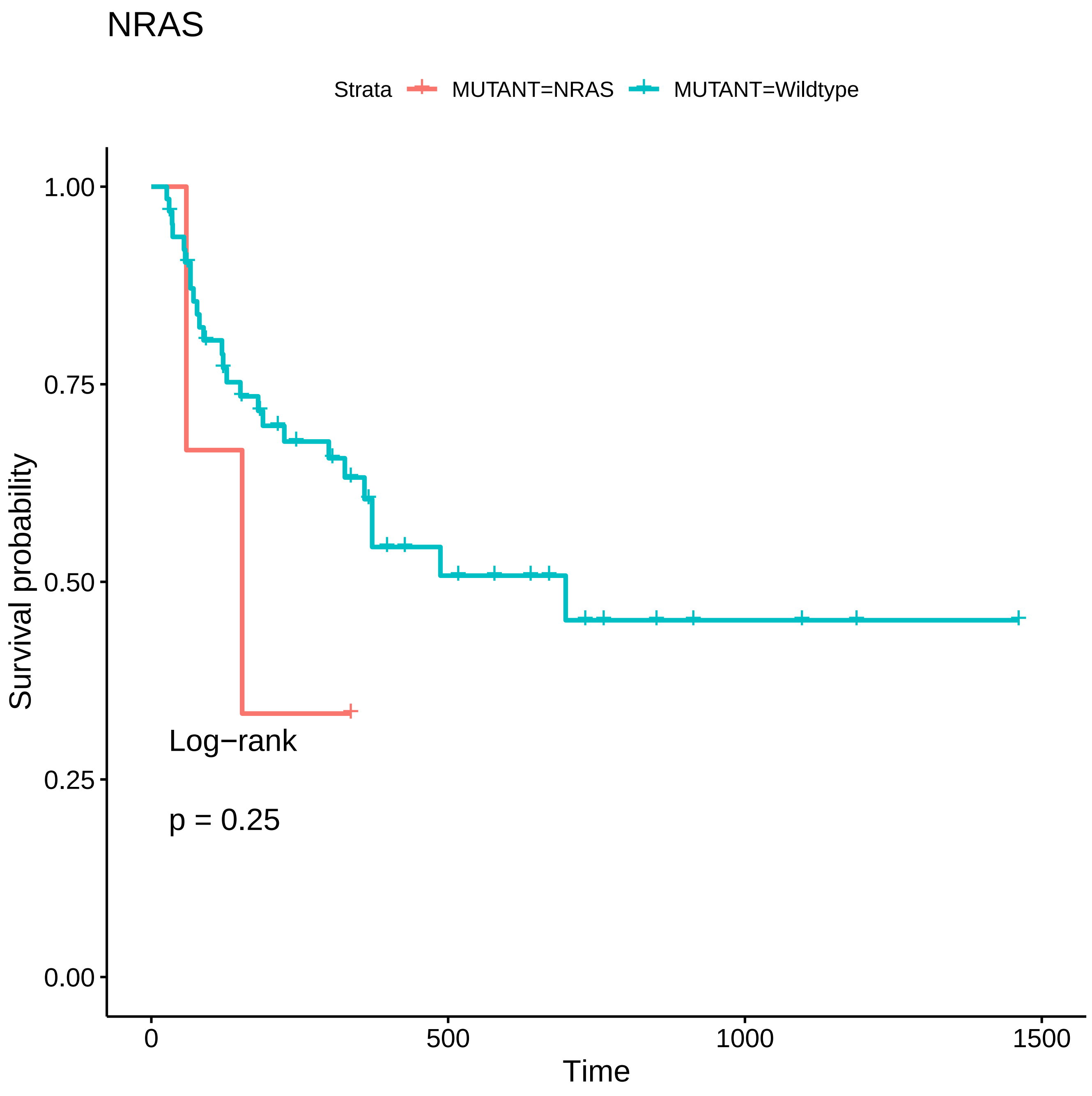
NRAS
Strata
MUTANT=NRAS
MUTANT=Wildtype
Survival probability
1.00
0.75
0.50
0.25
0.00
Log−rank
p = 0.25
0
500
1000
1500
Time

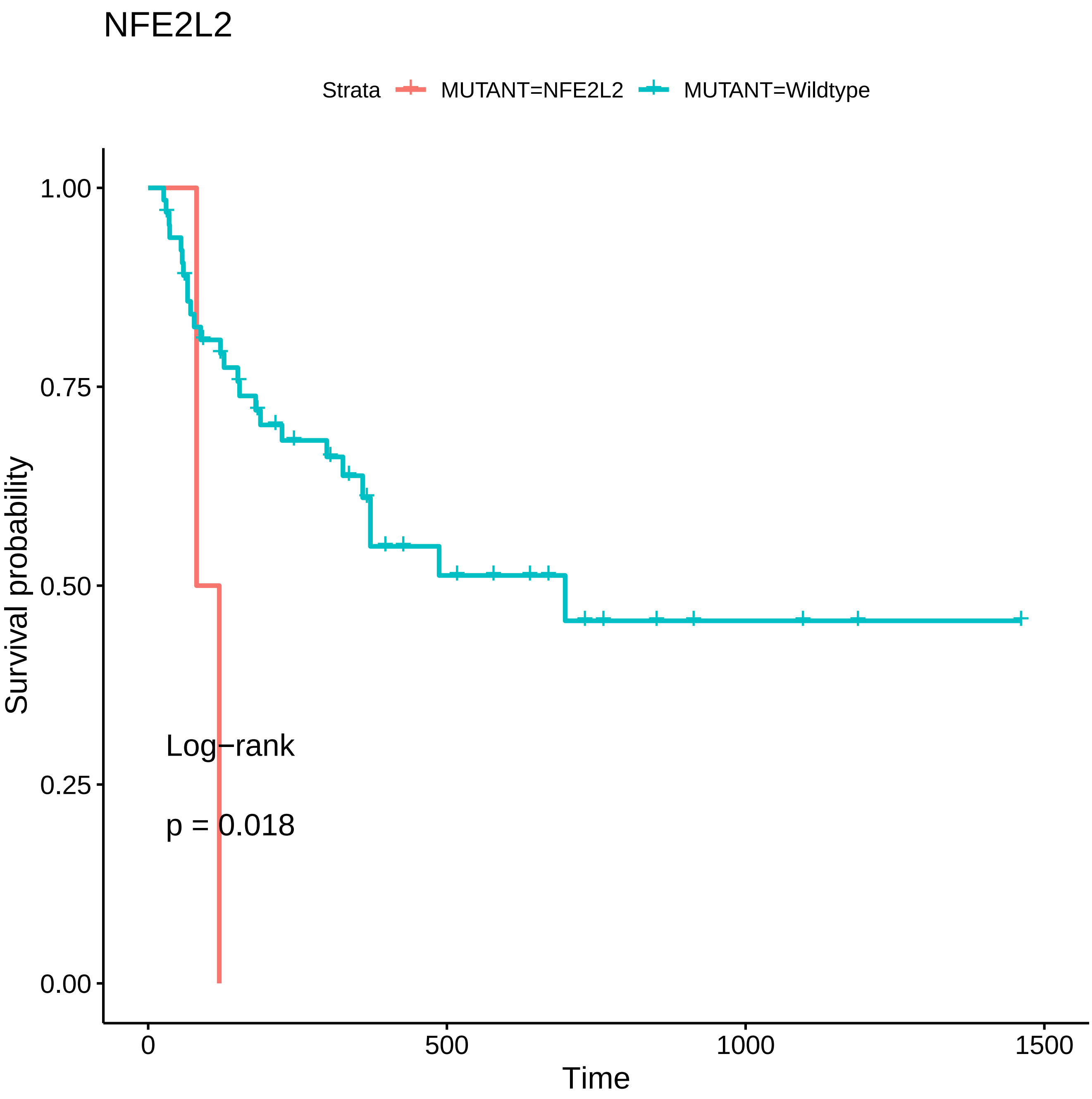
NFE2L2
Strata
MUTANT=NFE2L2
MUTANT=Wildtype
Survival probability
1.00
0.75
0.50
0.25
0.00
Log−rank
p = 0.018
0
500
1000
1500
Time

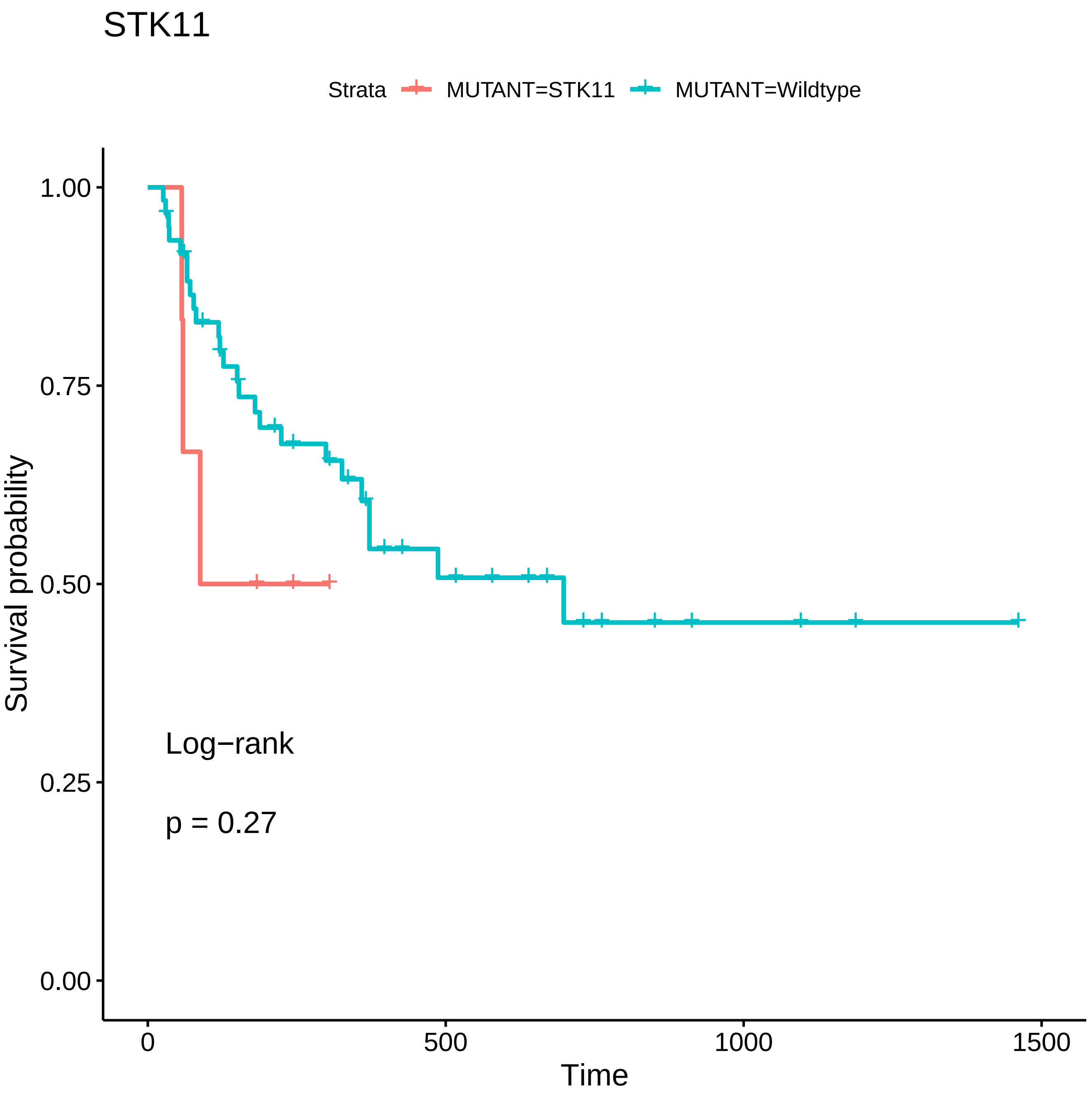

STK11
Strata
MUTANT=STK11
MUTANT=Wildtype
Survival probability
1.00
0.75
0.50
0.25
0.00
Log−rank
p = 0.27
0
500
1000
1500
Time

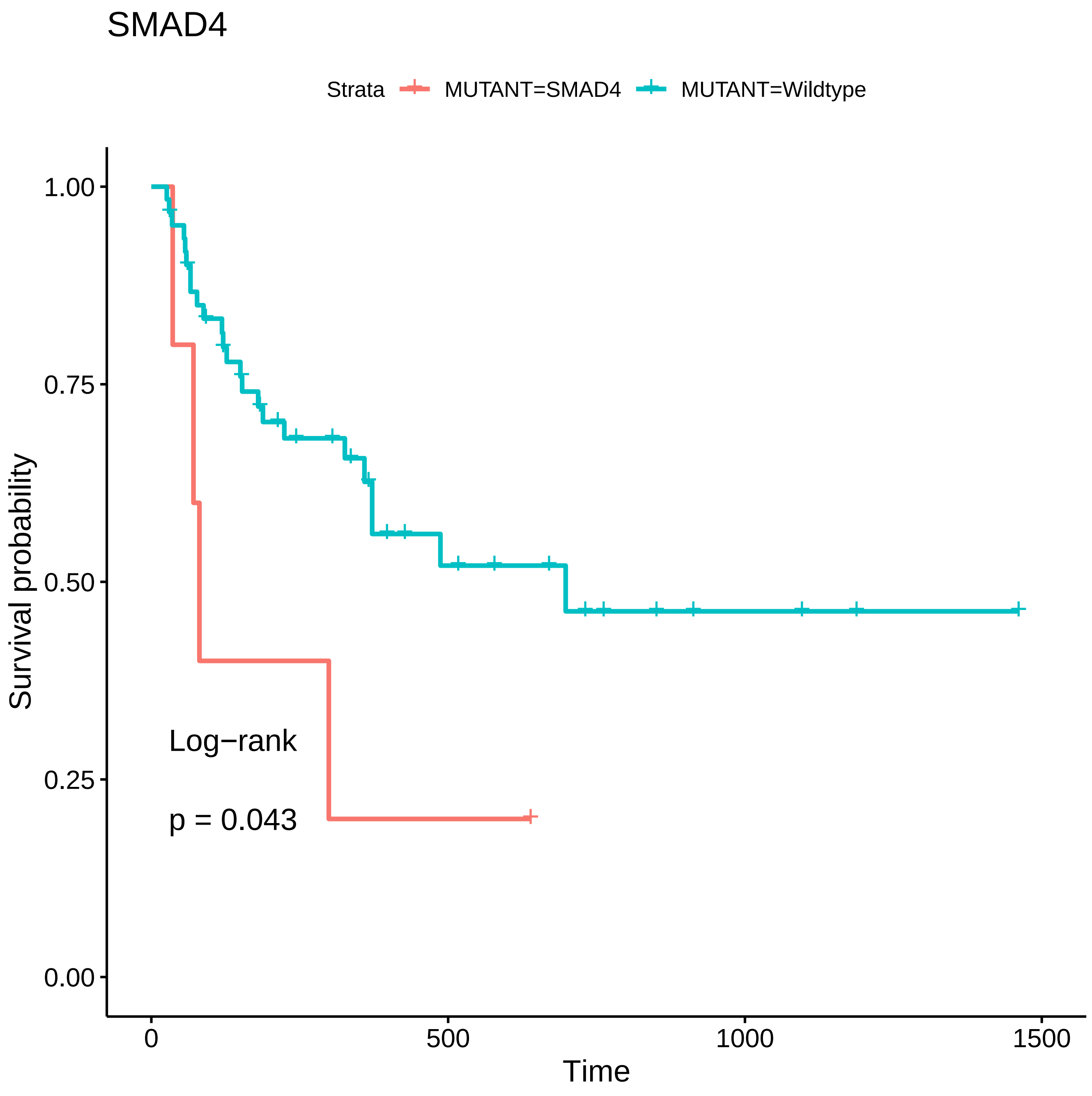
SMAD4
Strata
MUTANT=SMAD4
MUTANT=Wildtype
1.00
0.75
0.50
0.25
0.00
Survival probability
Log−rank
p = 0.043
0
500
1000
1500
Time

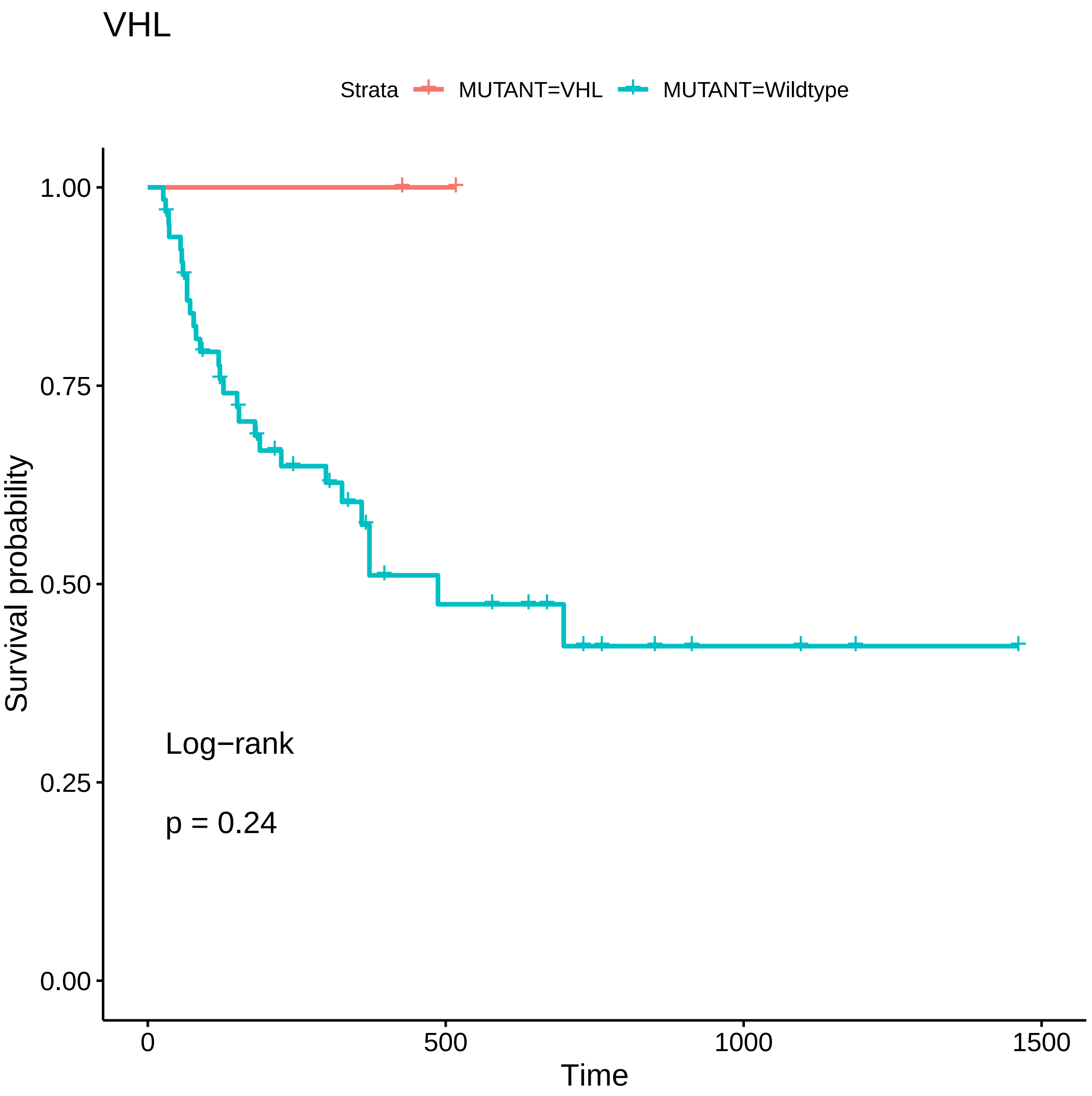

VHL
Strata
MUTANT=VHL
MUTANT=Wildtype
Survival probability
1.00
0.75
0.50
0.25
0.00
Log−rank
p = 0.24
0
500
1000
1500
Time

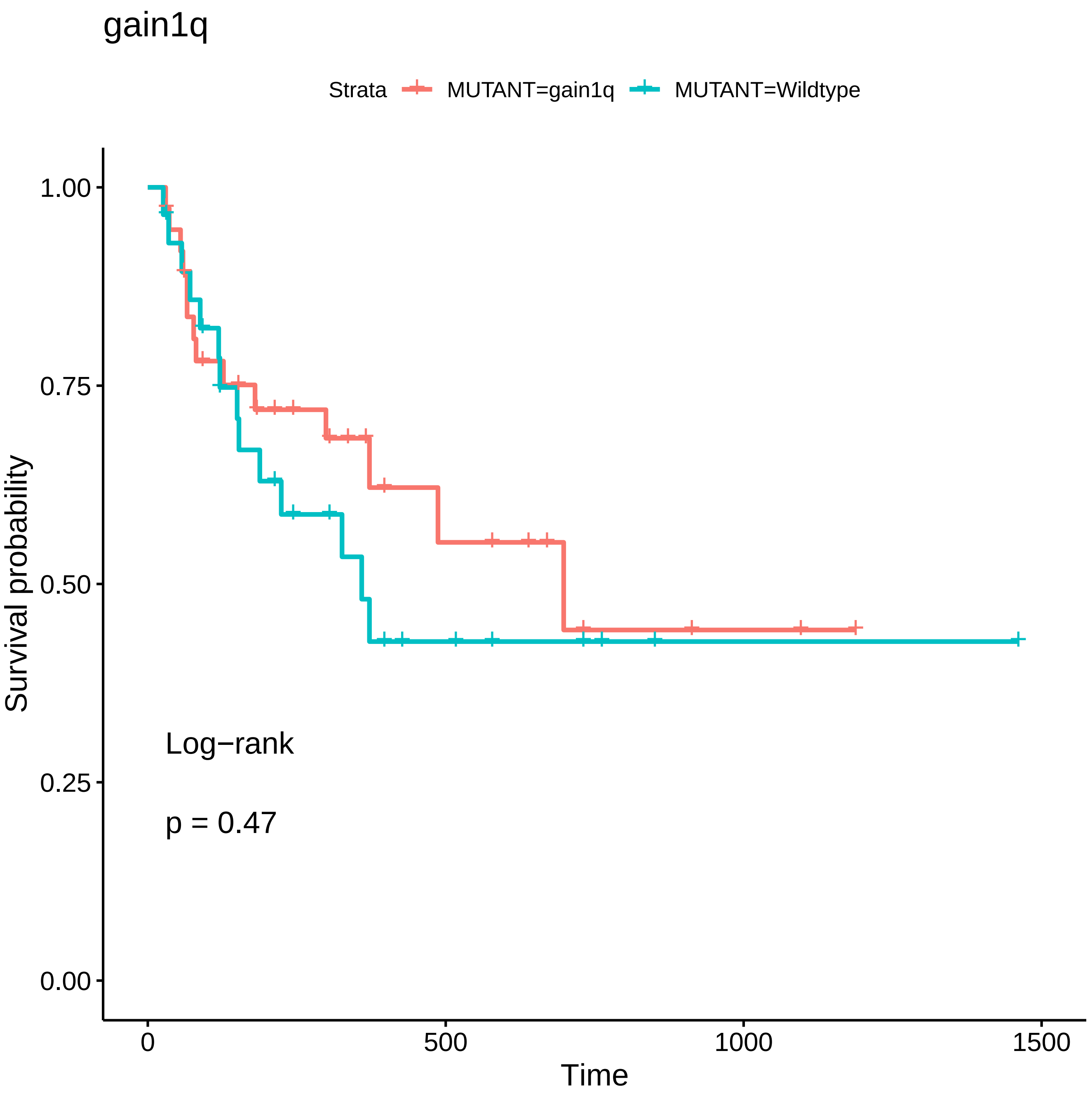

gain1q
Strata
MUTANT=gain1q
MUTANT=Wildtype
Survival probability
1.00
0.75
0.50
0.25
0.00
Log−rank
p = 0.47
0
500
1000
1500
Time

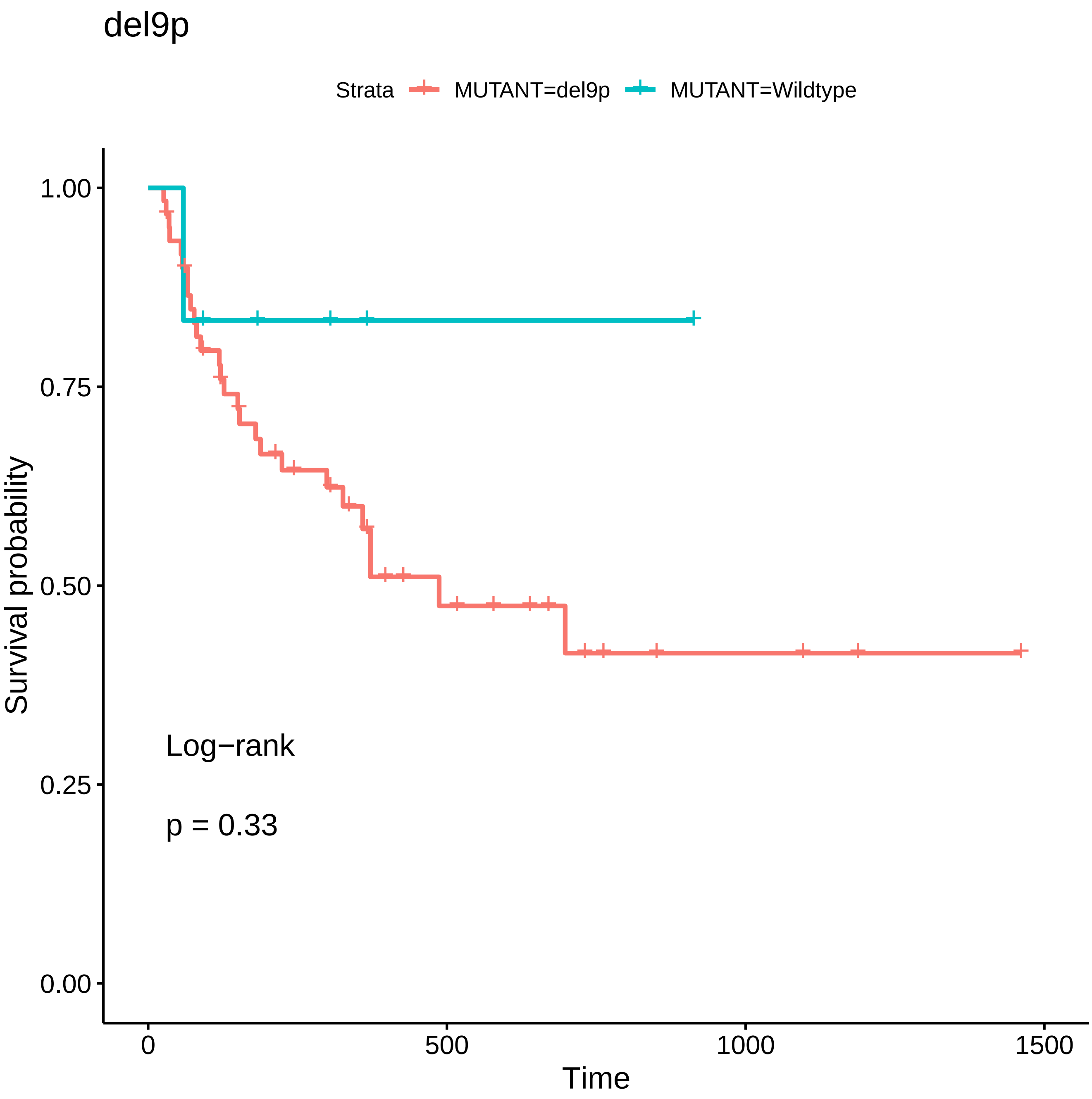

del9p
Strata
MUTANT=del9p
MUTANT=Wildtype
Survival probability
1.00
0.75
0.50
0.25
0.00
0
500
1000
1500
Time
Log−rank
p = 0.33

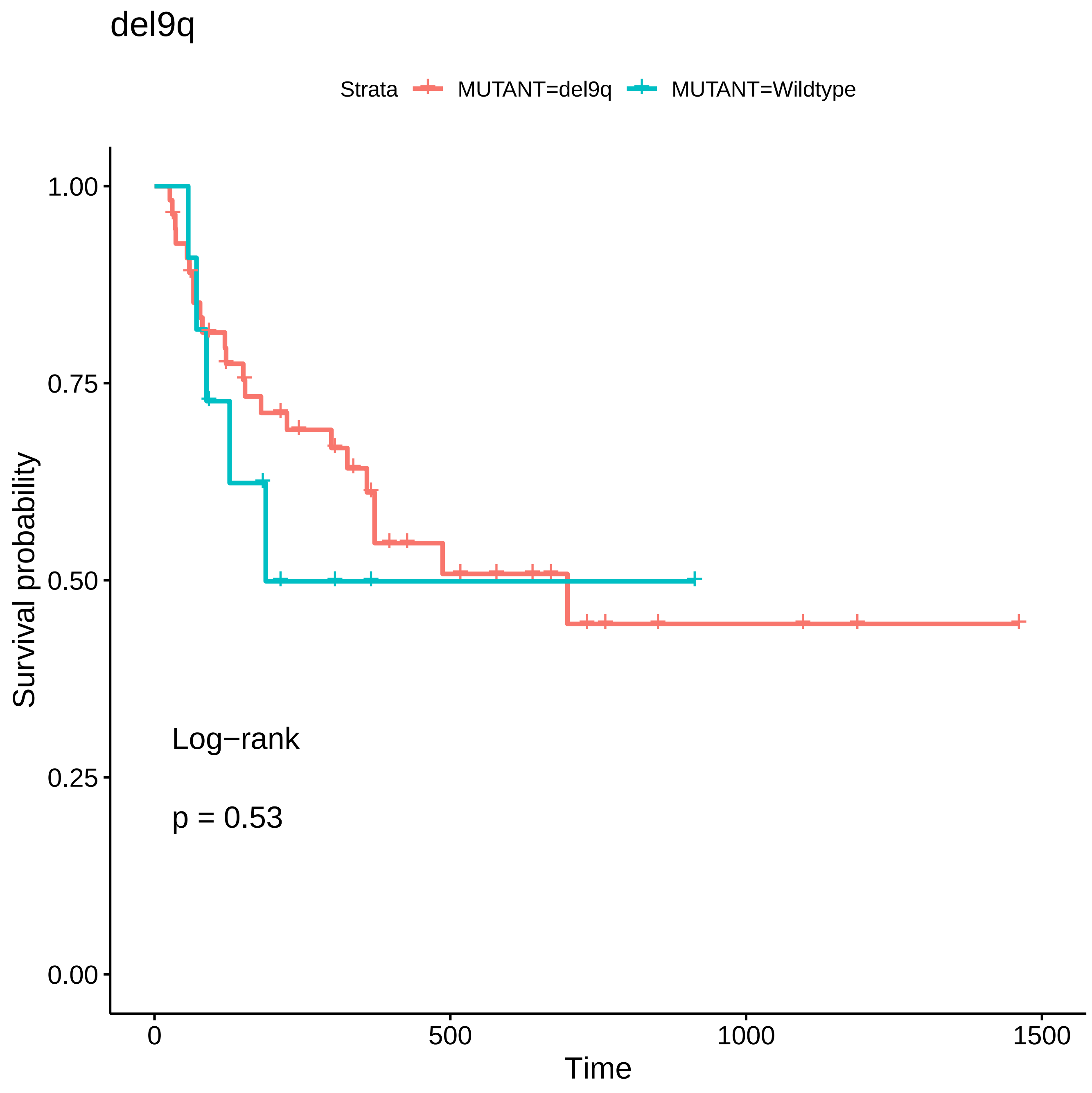
del9q
Strata
MUTANT=del9q
MUTANT=Wildtype
1.00
0.75
0.50
0.25
0.00
Survival probability
Log−rank
p = 0.53
0
500
1000
1500
Time

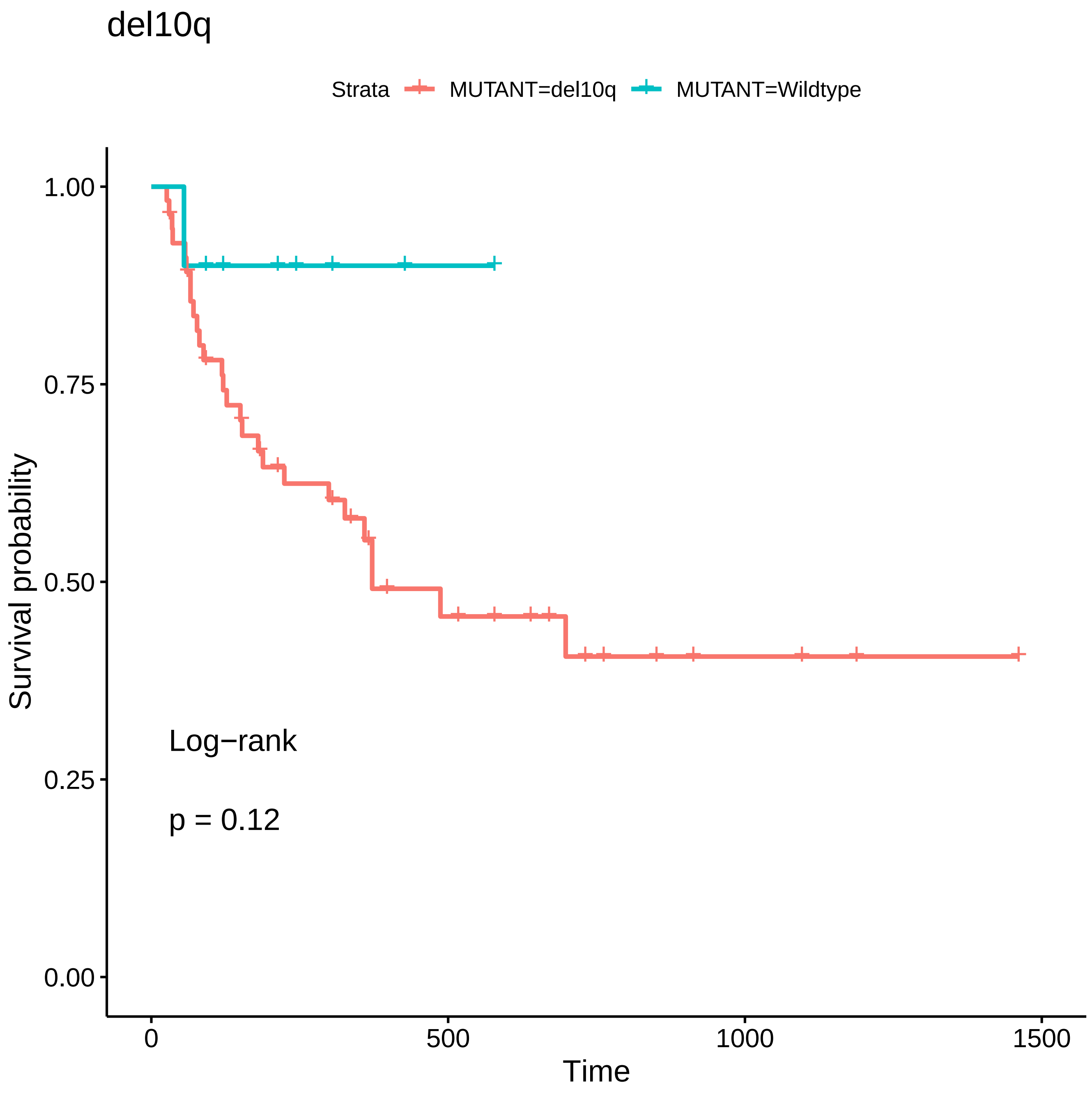

del10q
Strata
MUTANT=del10q
MUTANT=Wildtype
1.00
0.75
0.50
0.25
0.00
Survival probability
Log−rank
p = 0.12
0
500
1000
1500
Time

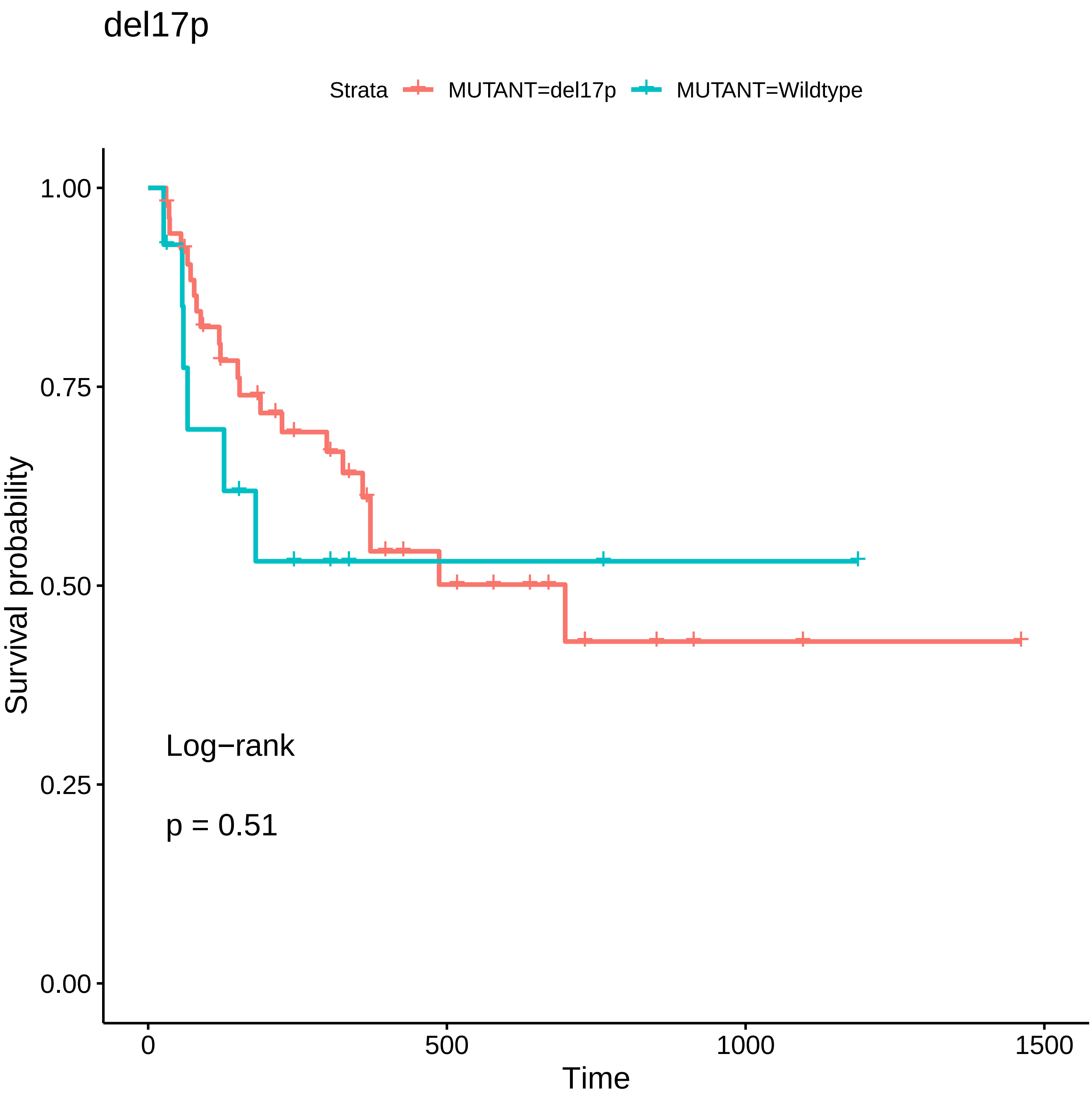
del17p
Strata
MUTANT=del17p
MUTANT=Wildtype
1.00
0.75
0.50
0.25
0.00
Survival probability
Log−rank
p = 0.51
0
500
1000
1500
Time

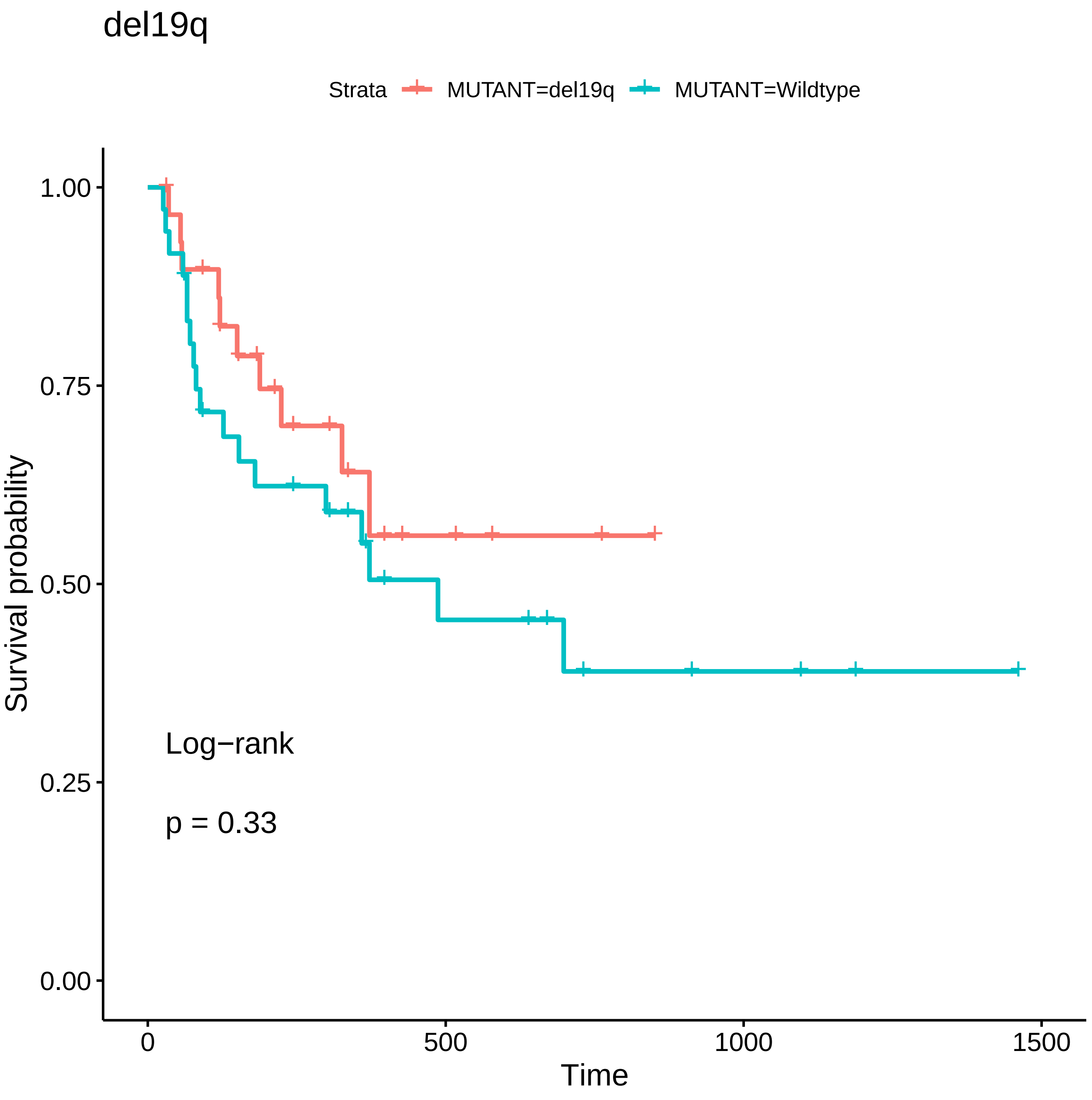
del19q
Strata
MUTANT=del19q
MUTANT=Wildtype
Survival probability
1.00
0.75
0.50
0.25
0.00
Log−rank
p = 0.33
0
500
1000
1500
Time

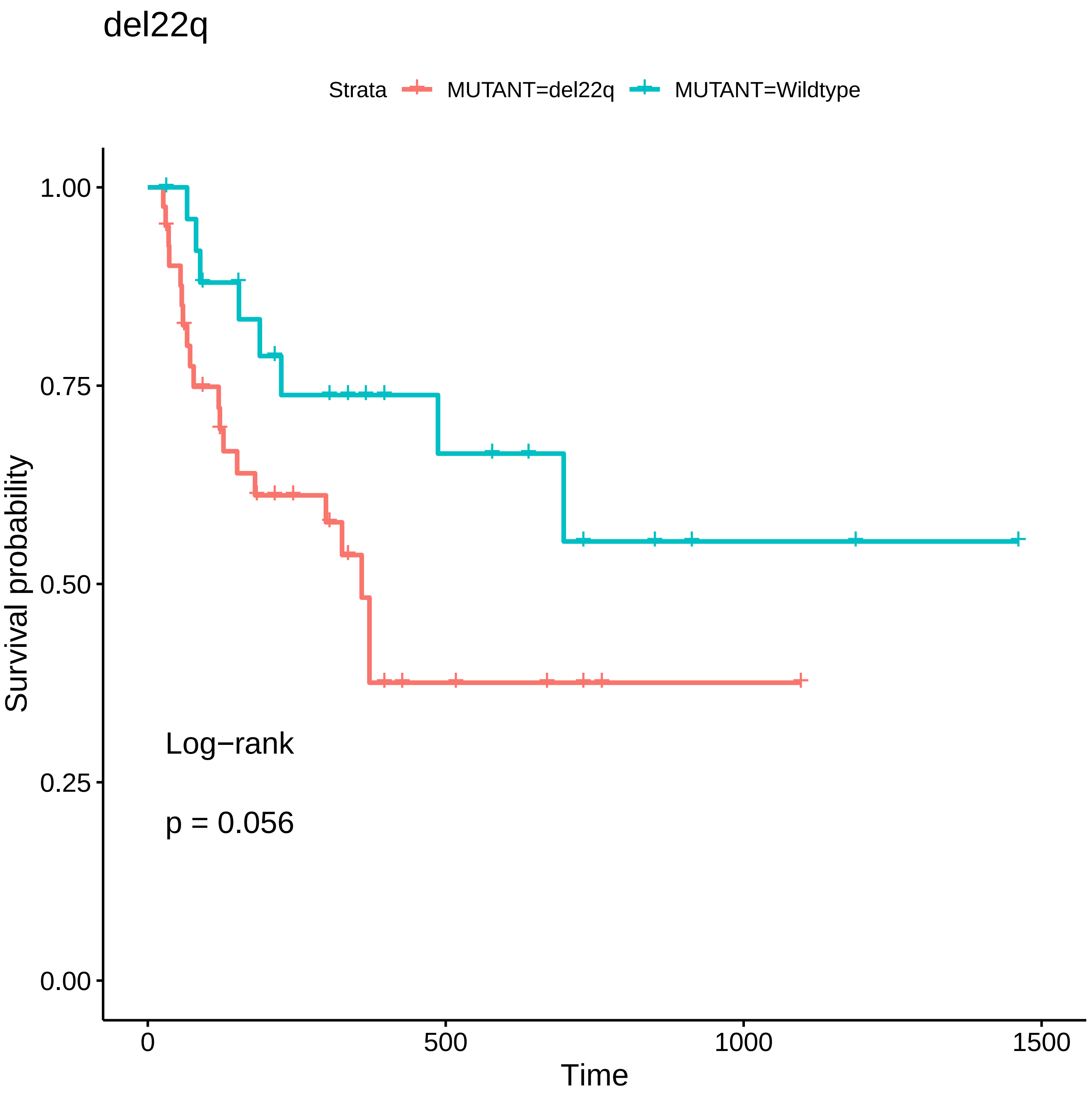
del22q
Strata
MUTANT=del22q
MUTANT=Wildtype
1.00
0.75
0.50
0.25
0.00
Survival probability
Log−rank
p = 0.056
0
500
1000
1500
Time

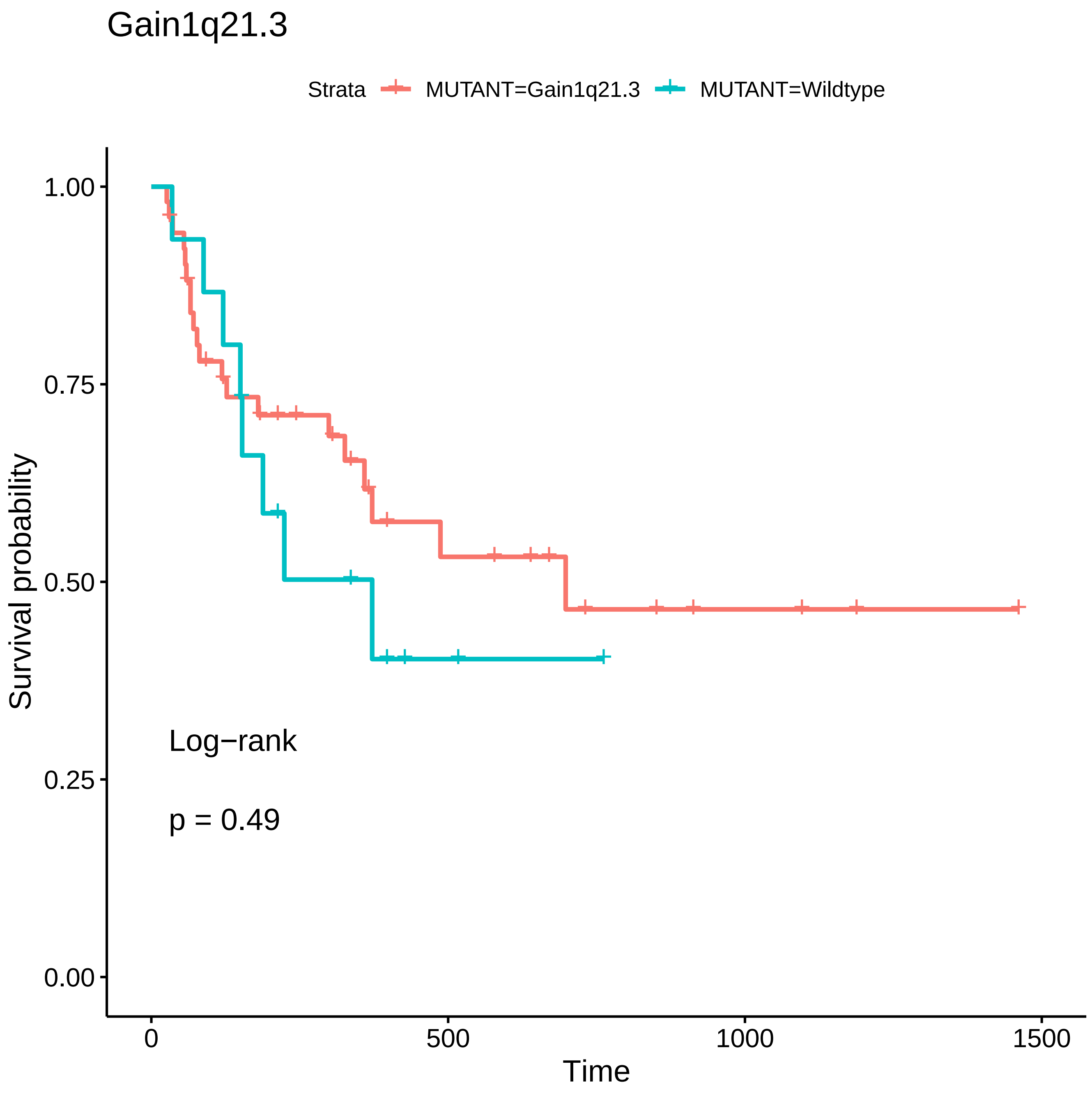
Gain1q21.3
Strata
MUTANT=Gain1q21.3
MUTANT=Wildtype
Survival probability
1.00
0.75
0.50
0.25
0.00
Log−rank
p = 0.49
0
500
1000
1500
Time

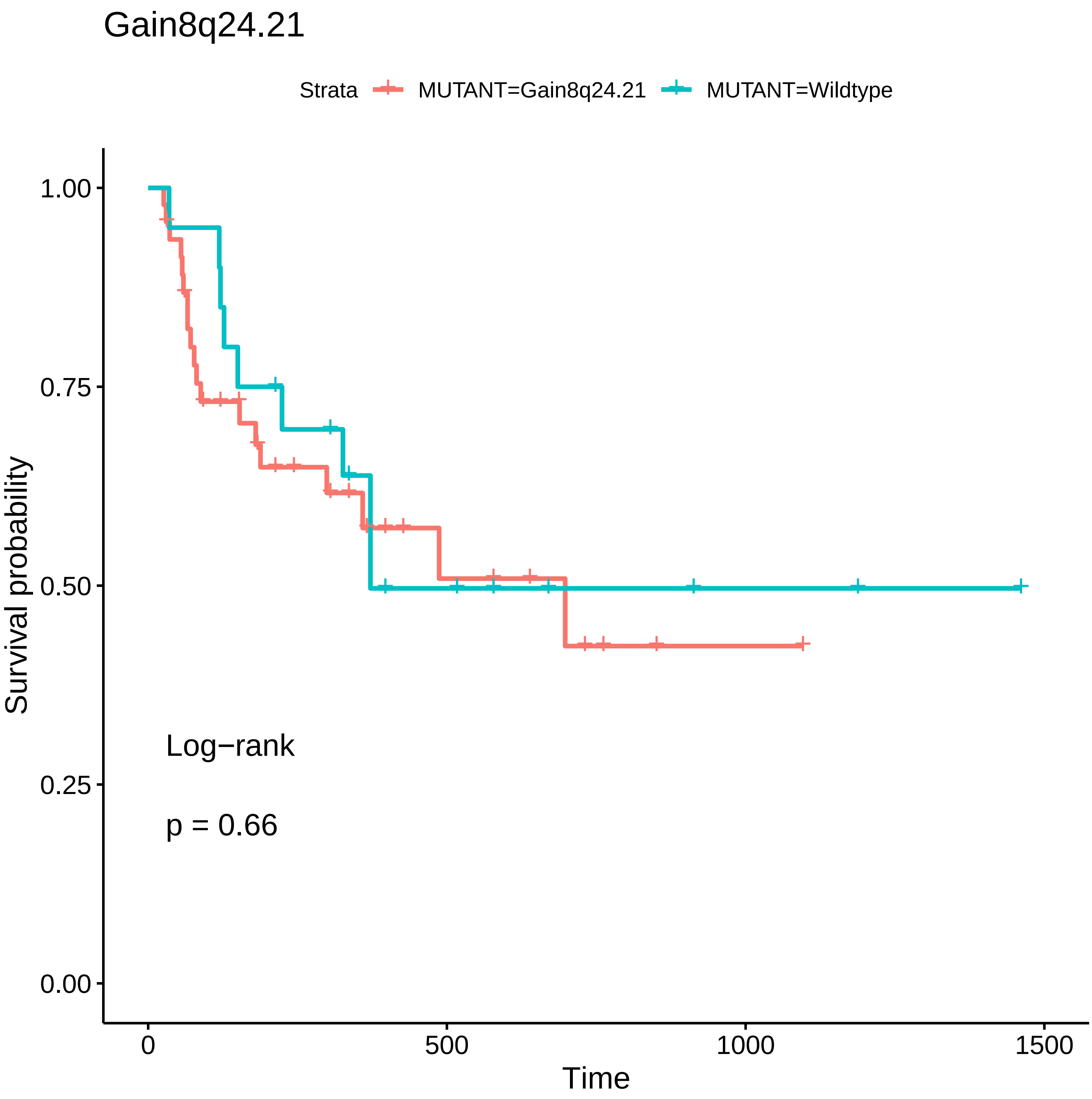

Gain8q24.21
Strata
MUTANT=Gain8q24.21
MUTANT=Wildtype
1.00
0.75
0.50
0.25
0.00
Survival probability
Log−rank
p = 0.66
0
500
1000
1500
Time

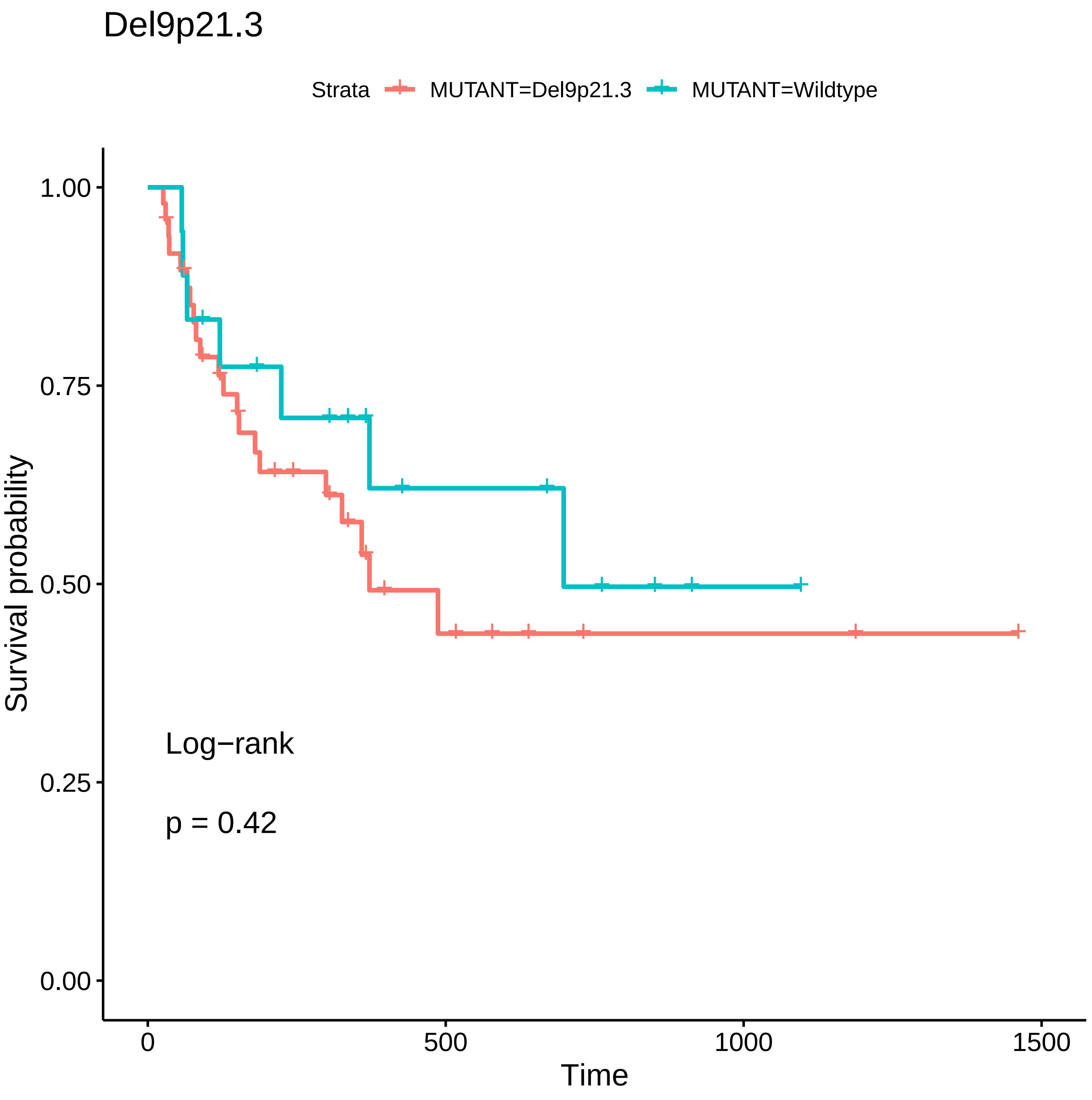

Del9p21.3
Strata
MUTANT=Del9p21.3
MUTANT=Wildtype
1.00
0.75
0.50
0.25
0.00
Survival probability
Log−rank
p = 0.42
0
500
1000
1500
Time

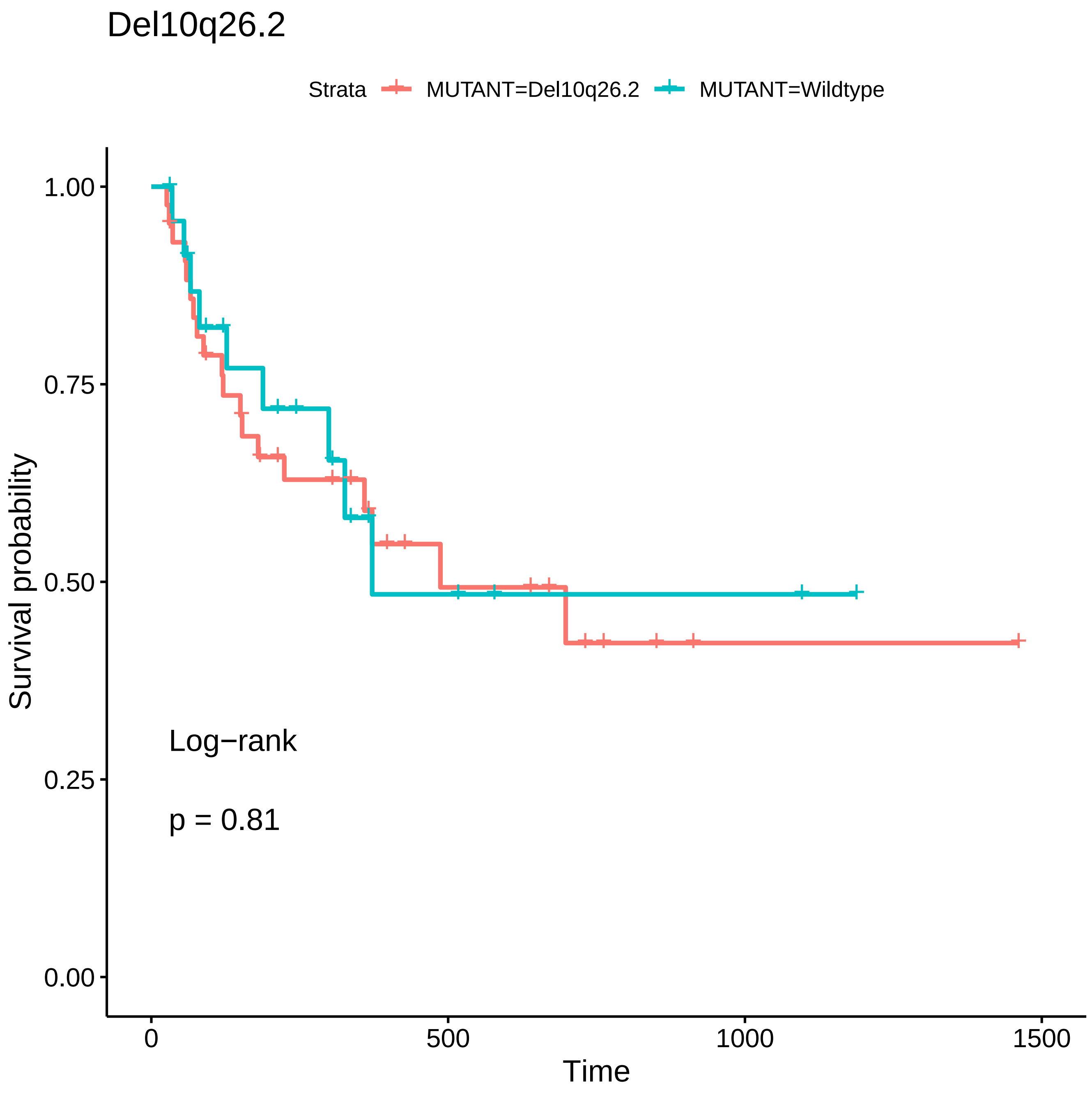

Del10q26.2
Strata
MUTANT=Del10q26.2
MUTANT=Wildtype
Survival probability
1.00
0.75
0.50
0.25
0.00
Log−rank
p = 0.81
0
500
1000
1500
Time

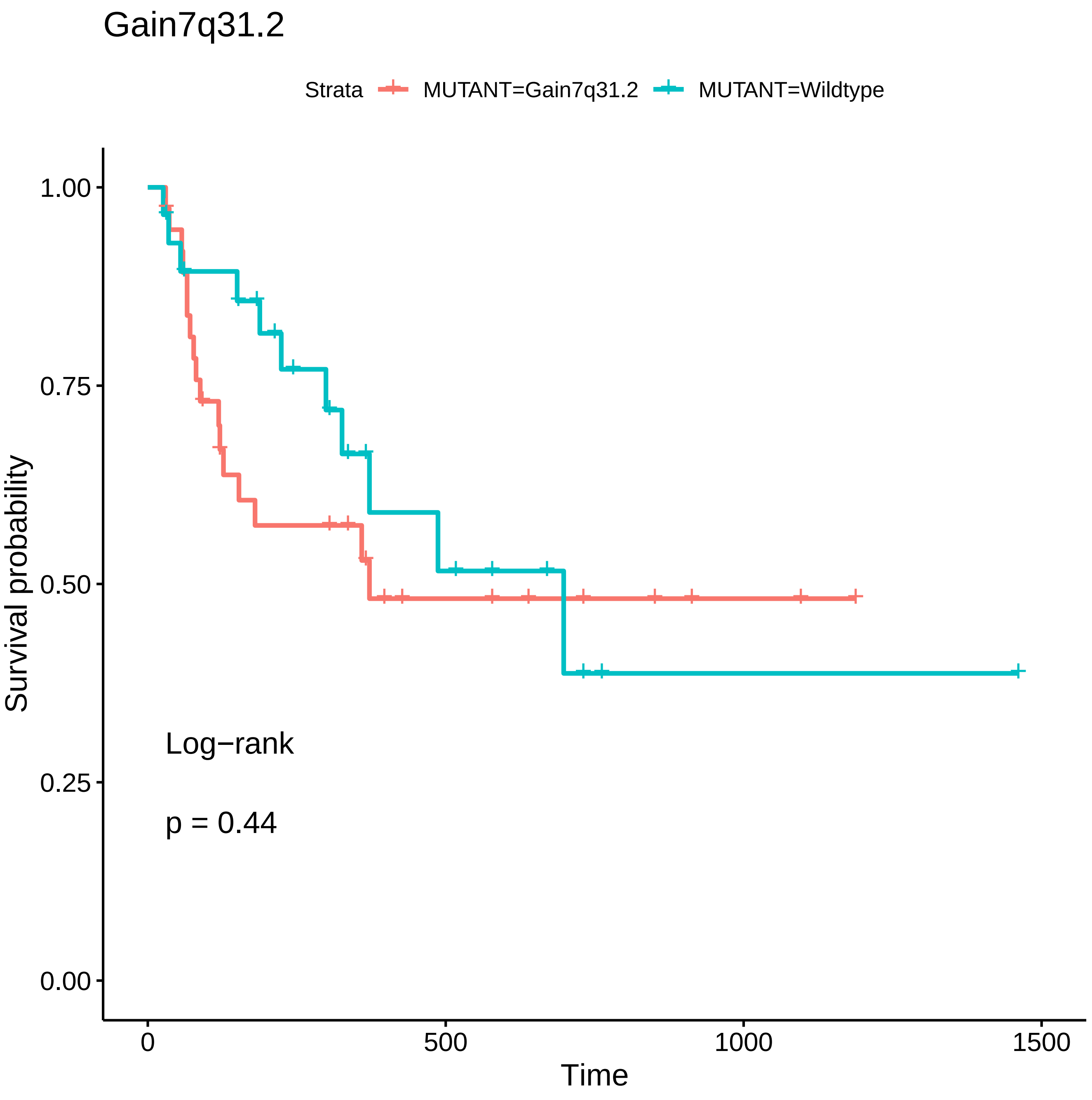
Gain7q31.2
Strata
MUTANT=Gain7q31.2
MUTANT=Wildtype
1.00
0.75
0.50
0.25
0.00
Survival probability
Log−rank
p = 0.44
0
500
1000
1500
Time

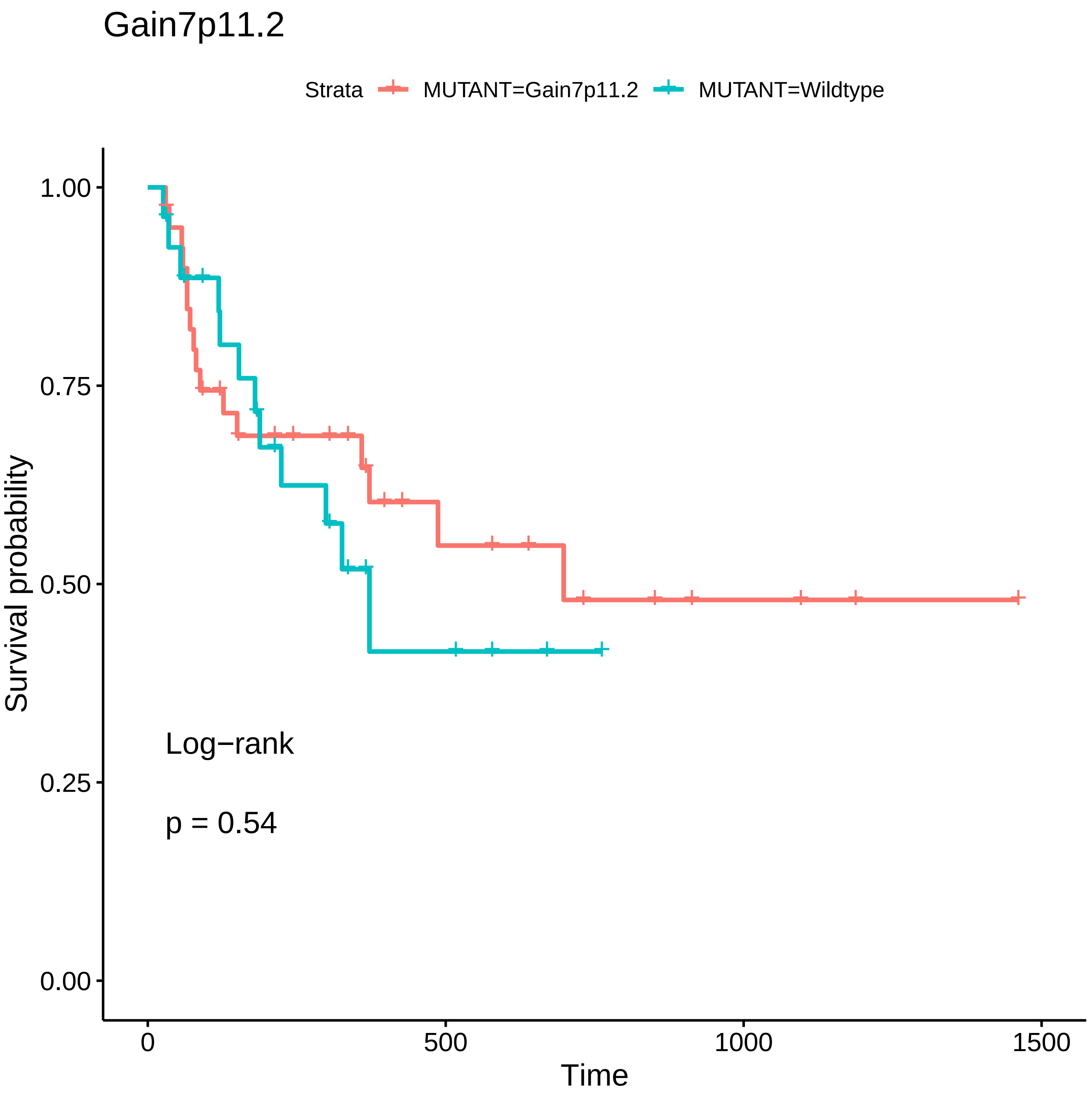
Gain7p11.2
Strata
MUTANT=Gain7p11.2
MUTANT=Wildtype
Survival probability
1.00
0.75
0.50
0.25
0.00
Log−rank
p = 0.54
0
500
1000
1500
Time

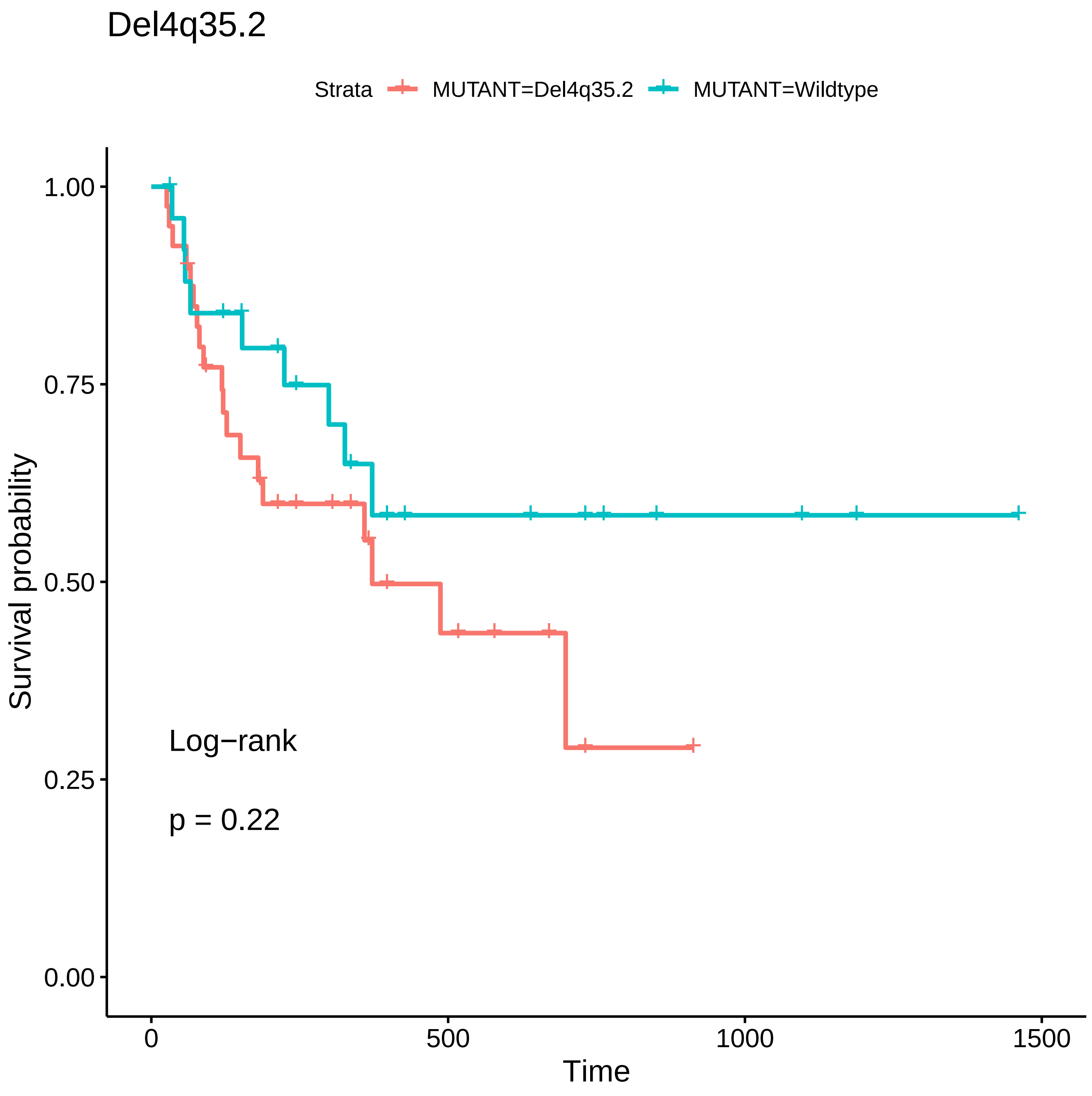

Del4q35.2
Strata
MUTANT=Del4q35.2
MUTANT=Wildtype
1.00
0.75
0.50
0.25
0.00
Survival probability
Log−rank
p = 0.22
0
500
1000
1500
Time

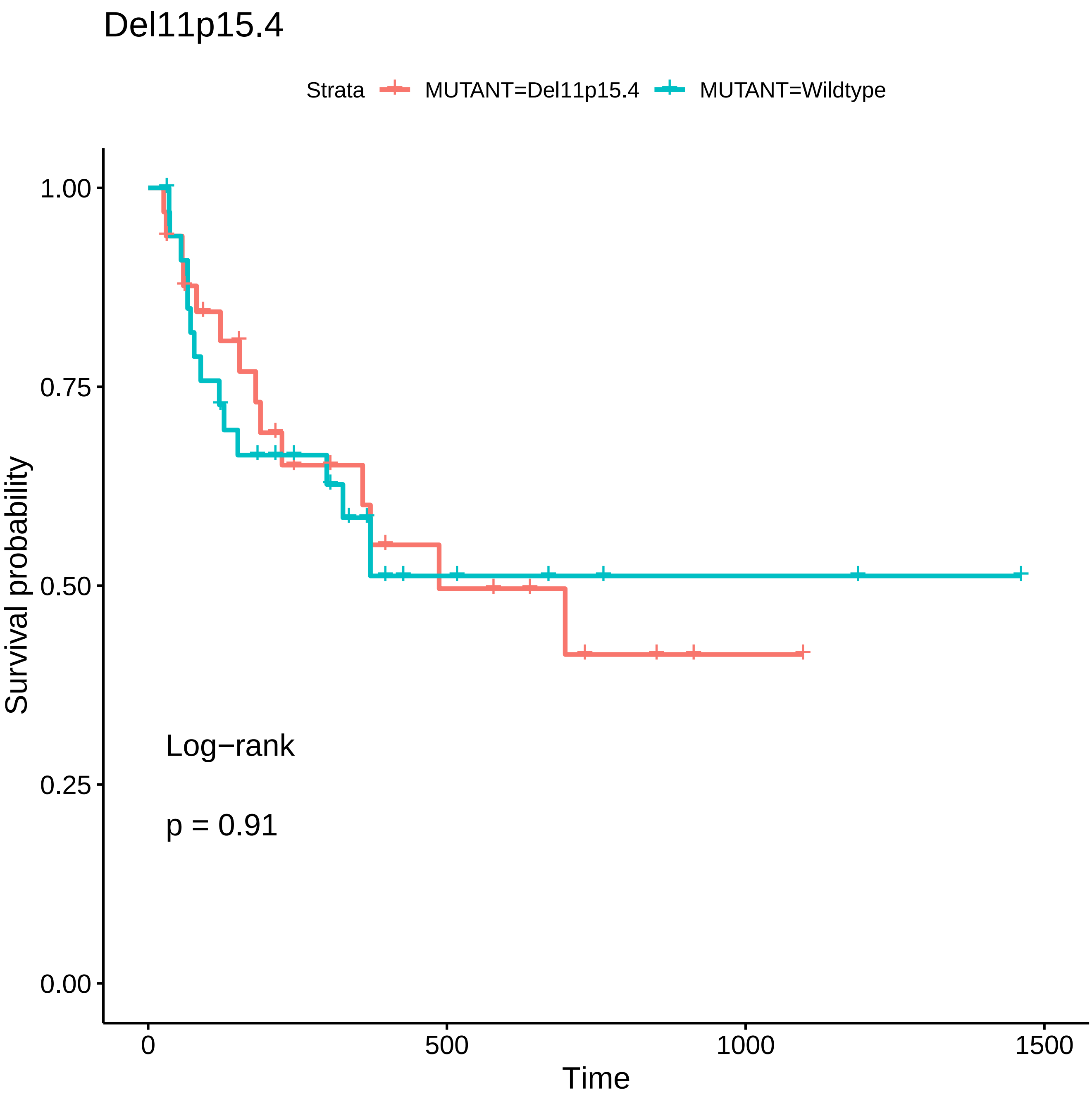

Del11p15.4
Strata
MUTANT=Del11p15.4
MUTANT=Wildtype
Survival probability
1.00
0.75
0.50
0.25
0.00
Log−rank
p = 0.91
0
500
1000
1500
Time

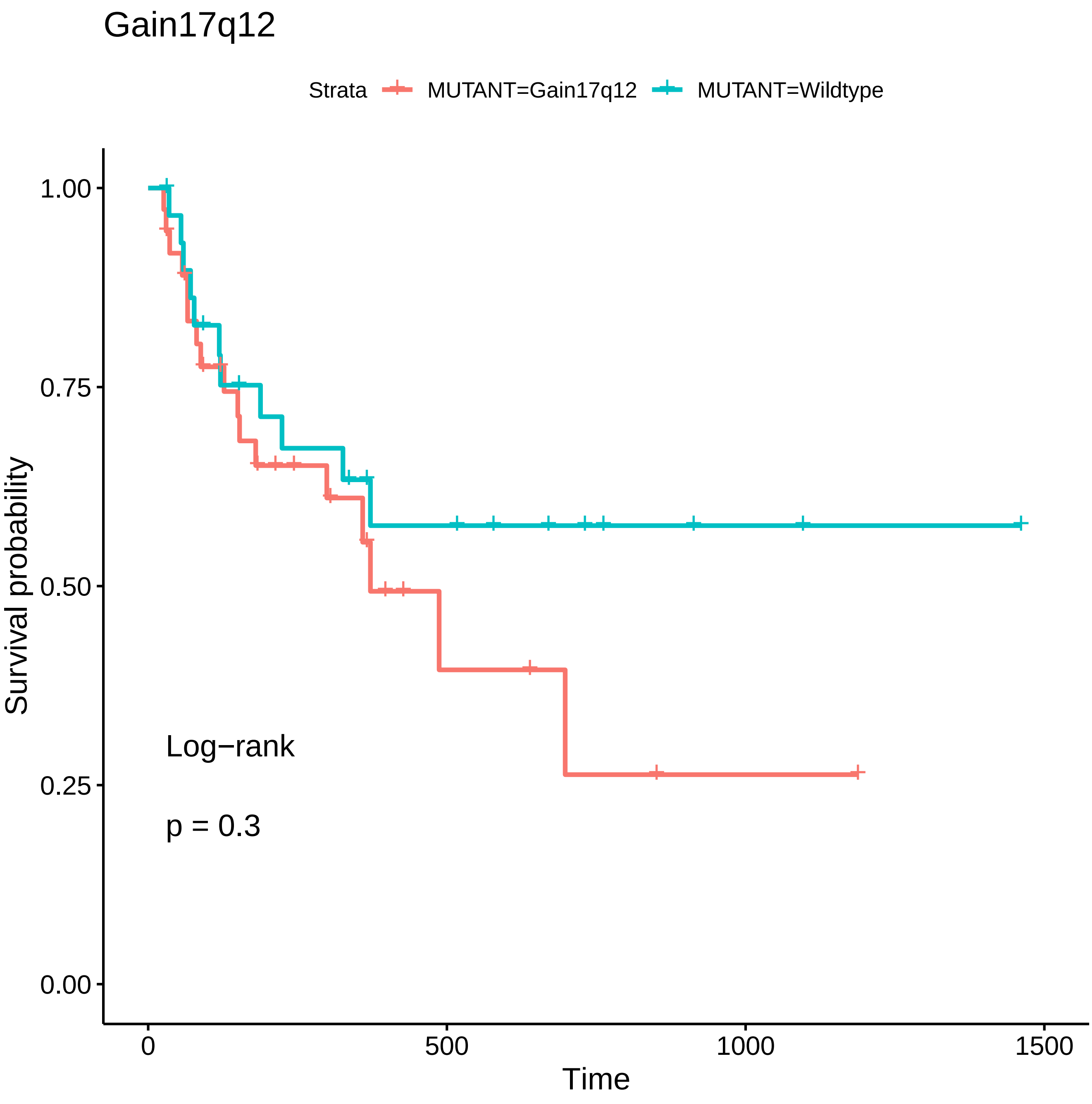
Gain17q12
Strata
MUTANT=Gain17q12
MUTANT=Wildtype
1.00
0.75
0.50
0.25
0.00
Survival probability
Log−rank
p = 0.3
0
500
1000
1500
Time

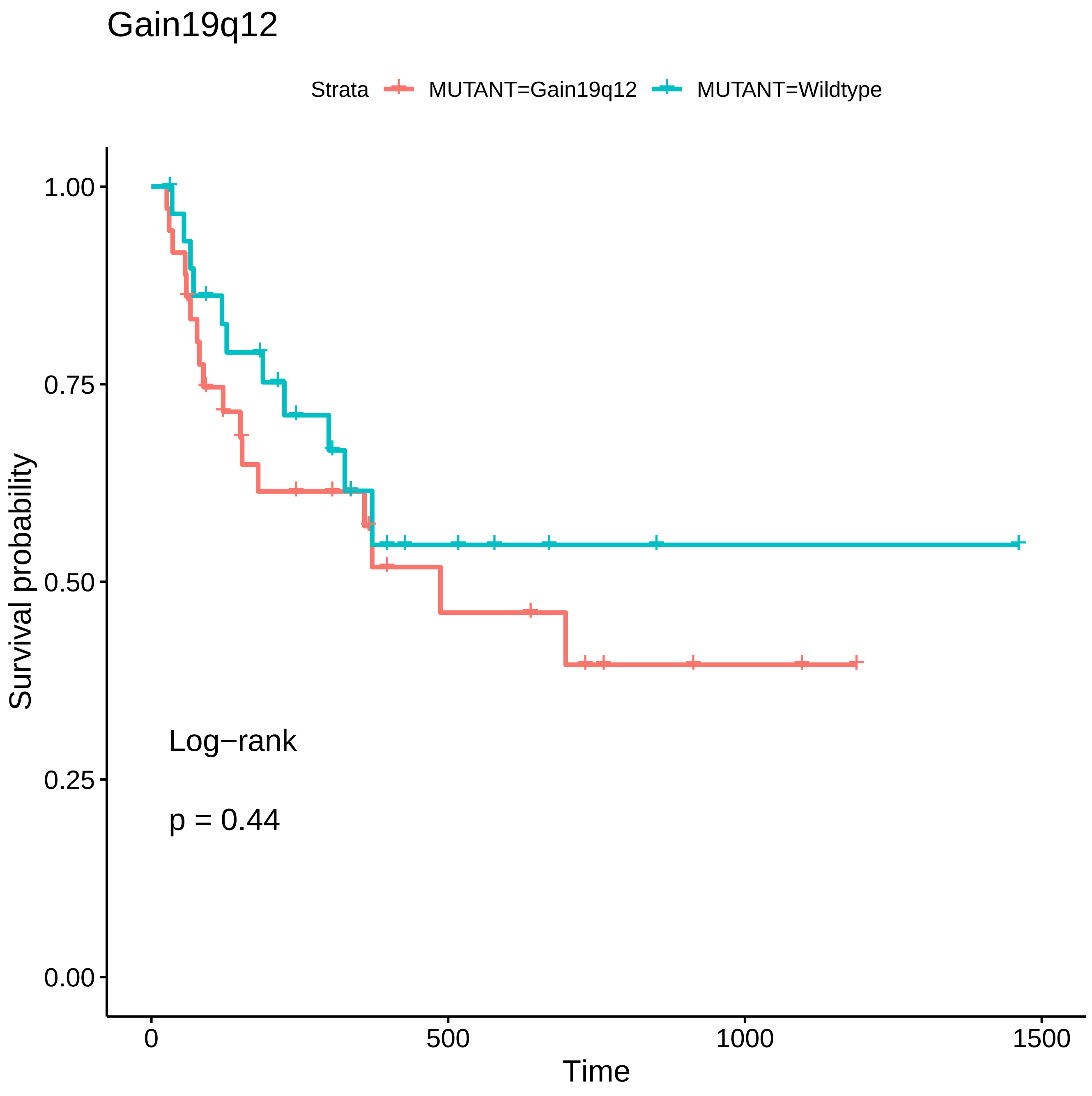
Gain19q12
Strata
MUTANT=Gain19q12
MUTANT=Wildtype
Survival probability
1.00
0.75
0.50
0.25
0.00
Log−rank
p = 0.44
0
500
1000
1500
Time

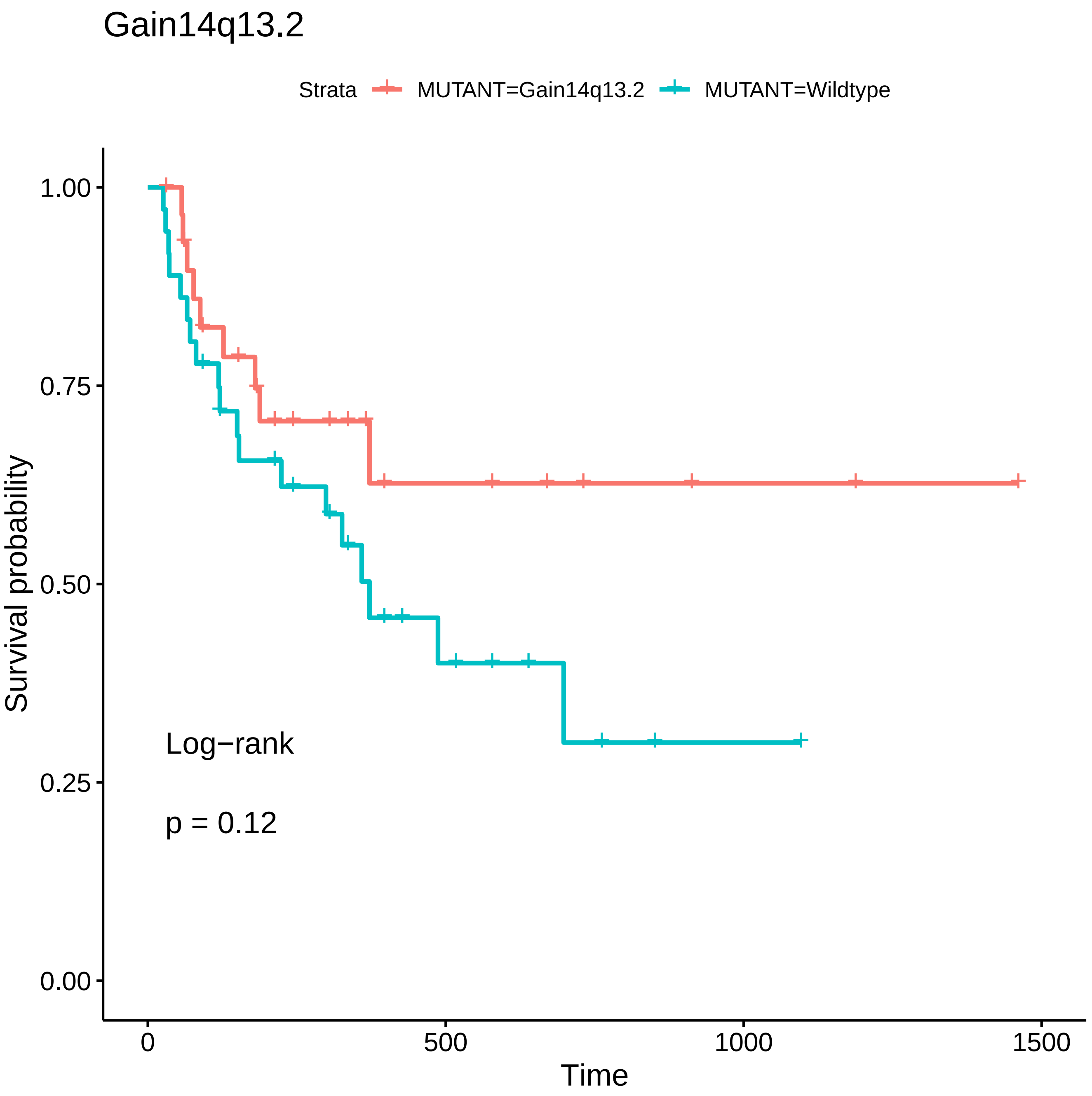
Gain14q13.2
Strata
MUTANT=Gain14q13.2
MUTANT=Wildtype
Survival probability
1.00
0.75
0.50
0.25
0.00
Log−rank
p = 0.12
0
500
1000
1500
Time

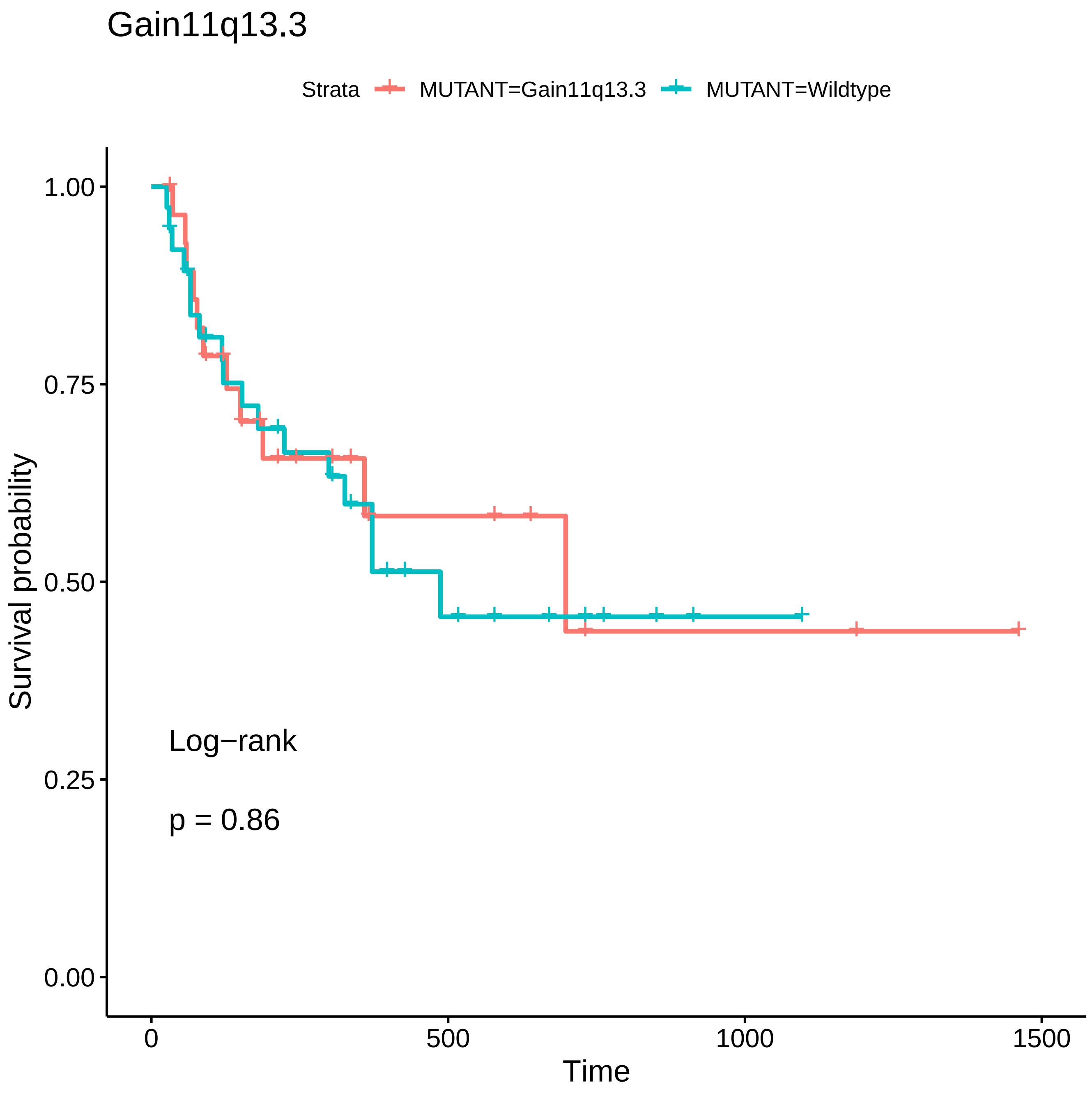
Gain11q13.3
Strata MUTANT=Gain11q13.3 MUTANT=Wildtype
Survival probability
1.00
0.75
0.50
0.25
0.00
0
500
1000
1500
Time
Log−rank
p = 0.86
